# Unjamming and emergent nonreciprocity in active ploughing through a compressible viscoelastic fluid

**DOI:** 10.1038/s41467-022-31984-z

**Published:** 2022-08-04

**Authors:** Jyoti Prasad Banerjee, Rituparno Mandal, Deb Sankar Banerjee, Shashi Thutupalli, Madan Rao

**Affiliations:** 1 Simons Centre for the Study of Living Machines, National Centre for Biological Sciences (TIFR), Bangalore, India; 2 Institute for Theoretical Physics, Georg-August-Universität Göttingen, 37077 Göttingen, Germany; 3 Department of Physics, Carnegie Mellon University, Pittsburgh, PA USA; 4 International Centre for Theoretical Sciences (TIFR), Bangalore, India

**Keywords:** Condensed-matter physics, Statistical physics, thermodynamics and nonlinear dynamics

## Abstract

A dilute suspension of active Brownian particles in a dense compressible viscoelastic fluid, forms a natural setting to study the emergence of nonreciprocity during a dynamical phase transition. At these densities, the transport of active particles is strongly influenced by the passive medium and shows a dynamical jamming transition as a function of activity and medium density. In the process, the compressible medium is actively churned up – for low activity, the active particle gets self-trapped in a cavity of its own making, while for large activity, the active particle ploughs through the medium, either accompanied by a moving anisotropic wake, or leaving a porous trail. A hydrodynamic approach makes it evident that the active particle generates a long-range density wake which breaks fore-aft symmetry, consistent with the simulations. Accounting for the back-reaction of the compressible medium leads to (i) dynamical jamming of the active particle, and (ii) a dynamical *non-reciprocal* attraction between two active particles moving along the same direction, with the trailing particle catching up with the leading one in finite time. We emphasize that these nonreciprocal effects appear only when the active particles are moving and so manifest in the vicinity of the jamming-unjamming transition.

## Abstract (web summary)

The field of dense active matter has been the fount of many intriguing phenomena. Here, authors show that nonreciprocal interactions can emerge between active particles due to a dynamical feedback between their motility and the corresponding slow remodelling of a dense passive compressible medium.

## Introduction

There is a lot of interest in the effective long-range interactions that emerge amongst active particles moving through a dynamically responsive medium. Examples include motile particles embedded in a Stokesian fluid^1^ or on an elastic substrate^2^ and diffusiophoretic flows in a viscous suspension of chemically active particles^3^. The absence of time reversal symmetry manifests in large density fluctuations at steady state^4,5^, and in the appearance of nonreciprocal interactions between particles^2,3,6–12^.

In this paper, we ask whether nonreciprocal interactions could emerge as a result of a dynamical phase transition. To realise such emergent nonreciprocity, we study a dilute collection of motile active particles embedded in a dense compressible fluid suspension close to dynamical arrest. Our results are based on (a) numerical simulations of an agent-based model, and (b) analytical and numerical treatments of hydrodynamic equations. We find that there is a dynamical feedback between the motility of the active particles and the corresponding slow remodelling of the passive compressible medium. Such active particles are *ploughers*, as opposed to *cruisers* whose motility speed is unaffected by the medium, e.g., ref. 2. As a result, ploughers exhibit a jamming-unjamming transition at fixed medium density. We find that in the unjammed phase, the moving active particles develop a dynamic nonreciprocal interaction with each other arising from the compressibility of the passive medium. We emphasise that this long-range nonreciprocal sensing appears only when the active particles are moving through a momentum non-conserving medium, and consequently shows up in the neighbourhood of the jamming-unjamming transition.

Our study reveals a hitherto unappreciated facet in this intensely researched field of dense active assemblies^13–21^, where the focus has been on fluidisation^15^, intermittency^16^ and jamming^16,17^ close to glass transition. The current work should be relevant to a variety of cellular and non-cellular contexts, where the medium is dense but compressible, pliable but slow to relax. Such situations can occur in the (i) transport of constituent or embedded particles in the cytoplasm^22,23^, (ii) facilitated transport of transcription factories and exogenous particles embedded within the cell nucleus^24^, (iii) movement of bacteria and cancer cells in fabricated soft porous media or in tissues^25–29^, (iv) burrowing movement of ants and worms in dense soil^30–33^, and (v) intrusion of active particles in a disordered bubble raft or a dense suspension of solid colloidal particles^34–36^.

## Results

### Dynamics of active Brownian particles in a passive medium

Our model of the two-dimensional background passive medium is similar to the Kob-Andersen^37,38^ binary mixture of soft spheres with a volume fraction *ϕ* so as to be able to tune it across a glass transition at constant temperature *T*. To this passive medium, we add a dilute amount *ϕ*_*a*_ ≪ *ϕ* of active Brownian soft particles (ABPs)^39–42^, which are made motile by assigning to them independent random forces **f** = *f* **n**, whose orientation ${{{{{{{\bf{n}}}}}}}}\equiv (\cos \theta ,\; \sin \theta )$ is exponentially correlated over a persistence time *τ*. The dynamics of all the interacting particles labelled *i* are described by a Langevin equation subject to a thermal noise **ϑ** of zero mean and variance equal to 2*γk*_*B*_*T*, while the subset $i\in {{{{{{{\mathcal{A}}}}}}}}$ of ABPs are subject to additional active stochastic forces,

1 $$m{\ddot{{{{{{{{\bf{x}}}}}}}}}}_{i} 	=-\gamma {\dot{{{{{{{{\bf{x}}}}}}}}}}_{i}-{\partial }_{i}\mathop{\sum }\limits_{j\ne i}^{N}{V}_{ij}+f{{{{{{{{\bf{n}}}}}}}}}_{i}\,{{\mathbb{1}}}_{(i\in {{{{{{{\mathcal{A}}}}}}}})}+{{{\mbox{}}}{{{\bf{\vartheta}}}} {{\mbox{}}}}_{i} , \\ {\dot{\theta }}_{i} 	={\xi }_{i}\quad {{{{{{{\rm{for}}}}}}}}\,i\in {{{{{{{\mathcal{A}}}}}}}}.$$

where ${{\mathbb{1}}}_{(i\in {{{{{{{\mathcal{A}}}}}}}})}$ is the indicator function which ensures that the active forces are restricted to particles *i* in the active set ${{{{{{{\mathcal{A}}}}}}}}$. The orientation angle *θ*_*i*_ undergoes rotational diffusion described by an athermal noise *ξ*_*i*_, with zero mean and correlation $\langle {\xi }_{i}(t){\xi }_{j}(t^{\prime} )\rangle=2{\tau }^{-1}{\delta }_{ij}\delta (t-t^{\prime} )$. Its effect on the **x**_*i*_-dynamics is an exponentially correlated vectorial noise with correlation time *τ*, which being unrelated to the drag *γ*, violates the fluctuation-dissipation relation. The inter-particle potential *V*_*ij*_ is taken to be purely repulsive (inverse power law) with particle diameter *σ* (details of the simulation appear in Supplementary Note 1). We work in the high friction limit, where particle inertia can be ignored.

Starting from homogeneous and isotropic initial conditions, we evolve the system to its steady state by integrating over a time of order 10^2^ *τ*_*α*_, where *τ*_*α*_ is the density relaxation time, also called *α*-relaxation time (Supplementary Note 2). Throughout, we work in the low-temperature regime *T* = 0.5, 10^−1^, 10^−3^, over a wide range of densities $\phi \in \left[0.08 ,\, 0.97\right]$, and scan through a broad range of the active parameters *f* and *τ*.

### Interplay between active self-propulsion and viscoelasticity of the medium

**Fig. 1:**
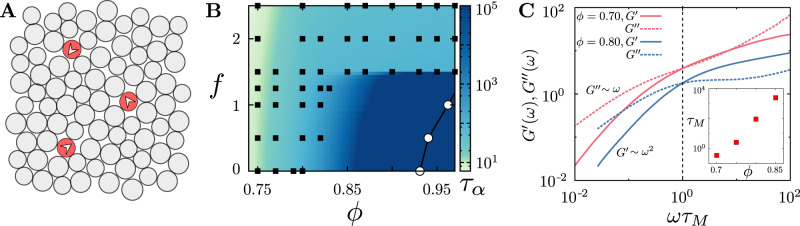
Active motile particles in a dense medium—approach to glass and viscoelasticity. **A** Schematic of dilute suspension of self-propelled particles of area fraction *ϕ*_*a*_ (red particles with arrows showing instantaneous direction **n** of propulsive force *f* **n**) moving through a dense compressible passive fluid of area fraction *ϕ* (grey particles). **B** Dynamical phase diagram in the *f* − *ϕ* plane for fixed *T* = 0.5, *ϕ*_*a*_ = 0.017 and *τ* = 50, showing macroscopic liquid and solid (glass) phases adjoining the cage-hopping “super-cooled liquid” regime, as determined from the *α*-relaxation time, *τ*_*α*_ (Supplementary Note 2). The glass transition at density *ϕ*_VFT_(*f*) (open circles) is obtained by fitting *τ*_*α*_ to a Vogel-Fulcher form (Supplementary Note 2). The black squares represent state-points where the simulations have been performed. **C** Frequency dependence of the elastic ${G}^{\prime}$ and viscous *G″* responses, at different values of *ϕ*, shows that the passive system (i.e., *f* = 0) behaves as a viscoelastic Maxwell fluid with relaxation time *τ*_*M*_. (inset) *τ*_*M*_ as a function of area fraction *ϕ* increases exponentially close to the glass transition. These quantities are time averaged (over the time origin) and ensemble averaged (over 64 independent simulations). The numerical errors are very small, less than 1% of the actual values.

Figure 1A shows a schematic of a dilute suspension (*ϕ*_*a*_ ≪ *ϕ*) of self-propelled particles moving through a dense compressible medium. While the macroscopic structural properties of such dense assemblies are rather innocuous, their dynamical features display characteristic slow relaxation, aging^43^ and dynamical arrest^15^ as the density *ϕ* is increased. The dynamics of the medium at large space and time scales, is summarised in a phase diagram (Fig. 1B) in the *f* − *ϕ* plane (for fixed *T*, *ϕ*_*a*_ and *τ*). The phase diagram is constructed by computing the *α*-relaxation time *τ*_*α*_ from the decay of the density overlap function *Q*(*t*) (Supplementary Note 2) using the definition *Q*(*τ*_*α*_) = 1/*e*. This phase diagram clearly shows macroscopic liquid and solid (glass) phases adjoining a cage-hopping “super-cooled” regime; fitting *τ*_*α*_ to a Vogel-Fulcher form (Supplementary Note 2) provides an estimate for the glass transition density *ϕ*_VFT_(*f*) (Fig. 1B)^15^.

Typical of an approach to a glass, the mean square displacement (MSD) averaged over all the passive particles shows a plateauing and cage-hopping dynamics, as the density *ϕ* is increased (Fig. S1)^34,44–46^. From these graphs we extract the long time diffusivity *D*_*∞*_ (Supplementary Note 2). In the limit *ϕ*_*a*_ ≪ *ϕ* and of small *τ*, we may deduce the linear microrheological properties of the passive medium from the Fourier transform of the MSD^47^, with an effective temperature obtained from the mean kinetic energy of the passive particles. Fig. 1C clearly shows that the medium is a viscoelastic Maxwell fluid, with the elastic response ${G}^{\prime} \sim {\omega }^{2}$ and the viscous response *G*^*″*^ ~ *ω*, for small *ω*, where the crossover timescale *τ*_*M*_ increases exponentially with the increase in area fraction *ϕ* close to the glass transition.

**Fig. 2:**
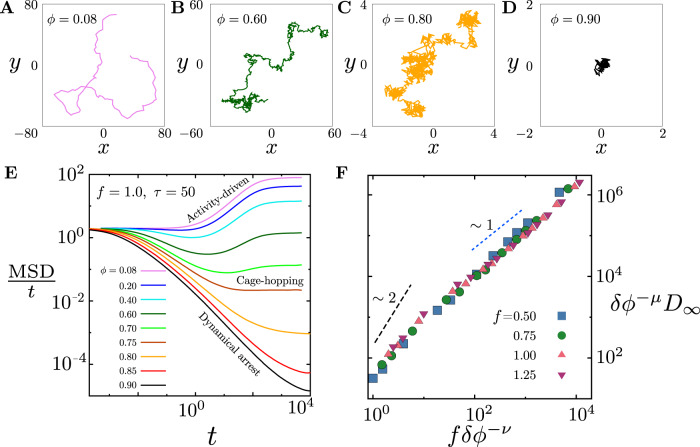
Crossover in transport characteristics of minority active particles. **A**–**D** Typical trajectories of the active particle as a function of *ϕ* at fixed *f* = 1 and *τ* = 50, showing (i) activity-dominated transport, (ii) glass dominated cage-hopping transport and (iii) dynamical arrest, recorded over a time *t* = 500. **E** Mean square displacement (scaled by time) computed from active particle trajectories suggests a crossover as a function of *ϕ*. These quantities are time averaged (over the time origin) and ensemble averaged (over 64 independent simulations). The numerical errors are very small, less than 1% of the actual values. **F** Crossover scaling collapse of the late time diffusion coefficient *D*_*∞*_(*ϕ*, *f*), described in (2), in the scaling variable *y* ≡ *f*/*δϕ*^*ν*^, where *δϕ* (0.026 ≤ *δϕ* ≤ 0.770) is the deviation from the MCT value, *ϕ*_MCT_(*f*). The scaling exponents are found to be *μ* ≈ 6.5 and *ν* ≈ 2.5. The dashed lines suggest a crossover of the scaling function from ${{{{{{{\mathcal{D}}}}}}}}(y)\to {y}^{2}$ to ${{{{{{{\mathcal{D}}}}}}}}(y)\to y$, as *y* increases.

We now turn our attention to the minority component, the small fraction of motile active particles—Fig. 2A–D show typical trajectories of the active motile particles at increasing values of *ϕ*, keeping  *f*  and *τ* fixed. The density of the passive medium affects the transport of the active particles—thus at low density *ϕ*, the motile particles show an *activity-dominated* transport (Fig. 2A, B), which crosses over to a *cage-hopping dominated* transport (Fig. 2C), as *ϕ* increases. As *ϕ* increases further, while still being less than *ϕ*_VFT_(*f*), the active particles get dynamically arrested, (Fig. 2D). The plot of the MSD of the active particles for the different values of *ϕ* (Fig. 2E), suggests a crossover collapse from activity-dominated diffusion proportional to *f*^2^*τ* to a glass dominated cage-hopping diffusion with a Vogel-Fulcher form to, finally, dynamical arrest. We verify this using a crossover scaling form for the late time diffusion coefficient (Fig. 2F)

2 $${D}_{\infty }(\phi ,\; f)=\delta {\phi }^{\mu }\,{{{{{{{\mathcal{D}}}}}}}}\left(y\equiv \frac{f}{\delta {\phi }^{\nu }}\right)$$

with *δϕ* = ϕ^*^(*f*) − *ϕ*, the deviation of *ϕ* from its value where the late time diffusion coefficient goes to zero. There are two possible choices for ϕ^*^(*f*)—the Vogel-Fulcher glass transition density *ϕ*_VFT_ (from an exponential fit) used to define the phase diagram in Fig. 1B and *ϕ*_MCT_, which is the mode-coupling estimate (having a power law form) of the glass transition. The reason for choosing *ϕ*_MCT_ = ϕ^*^(*f*) rather than *ϕ*_VFT_ is because the latter, being greater than *ϕ*_MCT_, is very difficult to approach either experimentally or in a simulation. The excellent collapse with exponents *μ* ≈ 6.5 and *ν* ≈ 2.5, suggests ‘critical behaviour’ at the mode-coupling transition, *ϕ*_MCT_(*f*). The asymptotic behaviour of the crossover scaling function ${{{{{{{\mathcal{D}}}}}}}}(y)$ at small *y* (Fig. 2F), suggests that *D*_*∞*_ ≈ *f*^2^ *δϕ*^3/2^, which crosses over to *D*_*∞*_ ≈ *f* *δϕ*^4^ as *y* = *fδϕ*^−2.5^ goes to *∞*.

We remark on the connection between the crossover scaling of the MSD of active particles as a function of the density of the passive medium with recent observations on the crossover behaviour of bacterial motility in a three dimensional porous medium as a function of porosity^25^. In as much as our study applies to this bacterial motility context, we suggest that the reported crossover in ref. 25 reflects a phenotypic change arising from a coupling of the normal bacterial movement to the physical properties of the dense passive medium.

### Remodelling of the compressible viscoelastic medium by the motile particles

We see that there is a strong feedback between the nature of active particle transport and the dynamical remodelling of the passive medium by the active particles^48^. This is especially prominent in the “super-cooled” liquid regime above the glass transition, where the active motile particles churn up the medium, inducing large density fluctuations that result in long-lived density modulations that back-react on the transport of the active particles. For a fixed active force *f* and temperature *T*, the physical characteristics of the under-dense regions are a result of the interplay between the active driving time *τ* and the *ϕ*-dependent density relaxation time, *τ*_*α*_(*ϕ*).

**Fig. 3:**
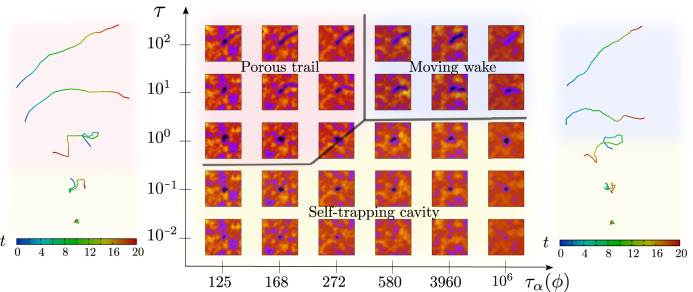
Remodelling of the compressible medium by the active particles. Under-dense regions in the compressible medium (darker blue colours indicate low *ρ*, and the yellow colours indicate a high *ρ* where *ρ* is the local density) that is remodelled by the motile particles, as a function of the persistence time *τ* and density relaxation time *τ*_*α*_(*ϕ*), for fixed (large) *f*. The geometry of the under-dense regions goes from being a static cavity to a moving wake around the motile particle, to a long-lived porous trail. The associated trajectories of the active particles at *τ*_*α*_(*ϕ*) = 168 (left of the phase diagram) and *τ*_*α*_(*ϕ*) = 3960 (right of the phase diagram) for the five values of *τ* corresponding to the phase diagram. The background colours outlining the particle trajectories corresponding to the respective regions marked on the phase diagram.

**Fig. 4:**
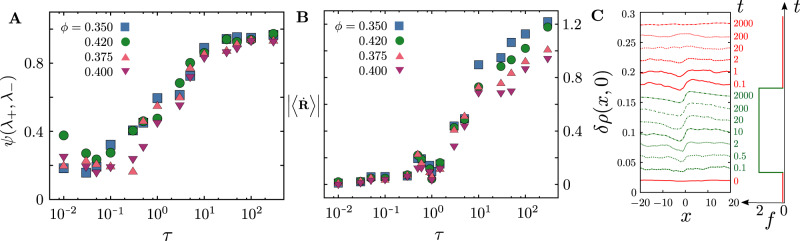
Dynamical transition in the active particle transport. **A** The geometry of the under-dense regions is characterised by a shape parameter, $\psi=\frac{{\lambda }_{+}-{\lambda }_{-}}{{\lambda }_{+}+{\lambda }_{-}}$, where *λ*_±_ are the eigenvalues of the moment of gyration tensor (Supplementary Note 3), and goes from being circular (*ψ* ≈ 0) to elongated (*ψ* ≈ 1) as *τ* increases. **B** Mean of the magnitude of the velocity (over a time interval Δ*t* = 20) of the active particle, as a function of the persistence time *τ* shows a dynamical transition at *τ* ≈ 1, below which it gets self-trapped in a cavity of its own making. **C** Dynamical response of the passive medium recorded at different time points, to a step active force from a single active particle (shown at right), measured in the frame of reference of the moving particle. The response is fore-aft asymmetric and relaxes slowly on switching off the active force.

Associated with a typical trajectory of the active particles shown in Fig. 3, we generate a density map of the medium in the vicinity of the active particle, as a function of *τ* and *τ*_*α*_(*ϕ*), keeping  *f*  large (*f* = 3.0) and *T* low (*T* = 10^−3^). The geometry and dynamics of the under-dense regions created by the active particles, show striking variations—(i) a halo (density wake) that moves with the motile particle, (ii) a static cavity that traps the active particle and (iii) a long-lived porous and tortuous trail as the active particle ploughs through the medium. In Fig. 4A, we show how the shape of the under-dense region sharply changes from circular to elongated as a function of *τ*. This geometrical transition appears to coincide with a dynamical transition in the active particle transport—Fig. 4B shows that the speed of the active particle $|\langle \dot{{{{{{{{\bf{R}}}}}}}}}\rangle|$ goes from being non-zero (where the active particle ploughs through the medium) to zero (where the active particle is self-trapped in a quasi-circular cavity of its own making), as *τ* decreases.

The profile and lifetime of the under-dense regions upon active remodelling, is a dynamical imprint of the transiting active particle (both its magnitude and direction) on the medium. In Fig. 4C, we activate only one of the particles of the medium, by imposing a step active force for a fixed duration. We measure the dynamical response of the passive medium (the change in *ρ*(*x*, *t*), the local density from its initial uniform profile) from the start of the activity, in the frame of reference of the moving active particle. We see that the density response *δρ*(*x*, *y*) is fore-aft asymmetric and that this asymmetric profile relaxes slowly on switching off the active force. This demonstrates that the passive medium (i) is a compressible fluid, and (ii) retains a memory of the moving event (its magnitude and direction) for some time.

In summary, we find that the active particles remodel the passive compressible medium and that the remodelled compressible medium reacts back on the active particle affecting its large scale movement. The back-reaction from the passive medium, can either facilitate movement of the active particle (in the ‘moving wake’ and ‘porous’ regimes) or trap the active particle (in the active ‘self-trapping’ regime—a similar self-trapping regime has been described in ref. 49, although it must be noted that the active particles considered there are squirmers, carrying a force-dipole in a momentum conserving background fluid. Self-propelled droplets have also recently been shown to get chemotactically caged in chemical trails of their own making^50^). A striking example of such facilitated transport of active particles in a compressible gel is the the ATP-dependent movement of transcription factories that move through the dense nuclear medium of cells^24^.

### Hydrodynamics of active particles moving in a compressible viscoelastic fluid

For a deeper understanding of the interplay between the movement of the active particles and the asymmetric dynamical response of the compressible passive medium, we construct a set of active hydrodynamic equations^5^ and analyse their solutions in simple situations.

The passive compressible fluid is described by the local density *ρ* and velocity **v** fields, while the dilute collection of active particles *i* with position **R**_*i*_(*t*) are propelled by an active force of magnitude *f* along their orientations **n**_*i*_(*t*). The density of the medium obeys a continuity equation,

3 $${\partial }_{t}\rho+\nabla \cdot \left(\rho {{{{{{{\bf{v}}}}}}}}\right)=0$$

Since the dynamics is overdamped, the local velocity of the medium is obtained by local force balance,

4 $${{\Gamma }}{{{{{{{\bf{v}}}}}}}}=\eta {\nabla }^{2}{{{{{{{\bf{v}}}}}}}}-B\rho \nabla \rho+\mathop{\sum}\limits_{i\in {{{{{{{\mathcal{A}}}}}}}}}f\,{{{{{{{{\bf{n}}}}}}}}}_{i}(t)\delta \left({{{{{{{\bf{r}}}}}}}}-{{{{{{{{\bf{R}}}}}}}}}_{i}(t)\right)$$

where the velocity of the compressible fluid is driven by the body-forces $f\,{{{{{{{{\bf{n}}}}}}}}}_{i\in {{{{{{{\mathcal{A}}}}}}}}}$ imposed by the moving active particles. Note that this contribution is present, since the dynamics takes place in a medium that does not conserve momentum.

The second term on the right represents the forces that oppose the movement of the passive particles that are being pushed by the active body-forces. These come from an active pressure, which, to leading order, arises from a local compressibility of the passive fluid, *P* ∝ *ρ*^2^ + …, due to inter-particle interactions (at low *T*, the linear contribution is insignificant). The “compressibility” *B*, with units of *force* × *length*, is positive and can in principle depend on *ρ*.

The other terms correspond to the usual momentum dissipation, a viscous contribution *η* coming from collisions with the passive particles and friction Γ arising from both collisions with other particles and from the ambient medium. Importantly, in the high density regime approaching the glass transition, these kinetic coefficients *η* and Γ may be strongly dependent on the local density *ρ*.

The dynamics of the active particles in the overdamped limit is also given by force balance. In the dilute limit, low *ϕ*_*a*_, when there are no direct interactions between active particles, the balance is again between the propulsive body-forces and the local pressure due to the compressible fluid,

5 $$\gamma {\dot{{{{{{{{\bf{R}}}}}}}}}}_{i}=\,f{{{{{{{{\bf{n}}}}}}}}}_{i}-C\rho \nabla \rho {\left|\right.}_{at\; {{{{{{{{\bf{R}}}}}}}}}_{i}}$$

6 $${\dot{\theta }}_{i}=\xi_{i}(t)$$

for all $i\in {{{{{{{\mathcal{A}}}}}}}}$. Note that ${{{\mathbf{n}}}}_i \equiv ( \cos \theta_i, \sin \theta_i)$, and the athermal orientational noise $\xi_i$ has zero mean and is delta-correlated, $\langle \xi_i(t) \xi_j(t') \rangle = 2 \tau^{-1} \delta_{ij} \delta(t-t')$. Equation (5) accounts for the back-reaction of the medium on the dynamics of the active particles, which feels a block due to particle pile up ahead of it. We have assumed in (6) that the direction of the propulsion force is set by some internal detailed-balance violating mechanism, independent of the passive medium. Note that the active compressibility *C* is positive, dependent on *ρ*, and could be different from *B*. Further, in the limit of low *ϕ*_*a*_, one expects *γ* to be a single particle friction, while Γ to be a collective frictional dissipation; the latter could be high when *ρ* is large.

We will refer to such polar active particles as *ploughers*, as opposed to *cruisers*, whose speed is unaffected by the medium, e.g., ref. 2.

Note that although the passive and active particles have comparable sizes, we treat the passive medium using a coarse-grained density, but the active particles as “point-particulate”. Thus, our hydrodynamic description should be valid over scales larger than a few particle sizes.

We now check whether the continuum hydrodynamic equations, (3)–(5), describe, in a coarse-grained sense, the agent-based dynamics represented by (17). For this we compute the local coarse-grained density and velocity fields of the passive fluid from our simulation trajectories, using an interpolation and smoothing scheme (Supplementary Note 3).

**Fig. 5:**
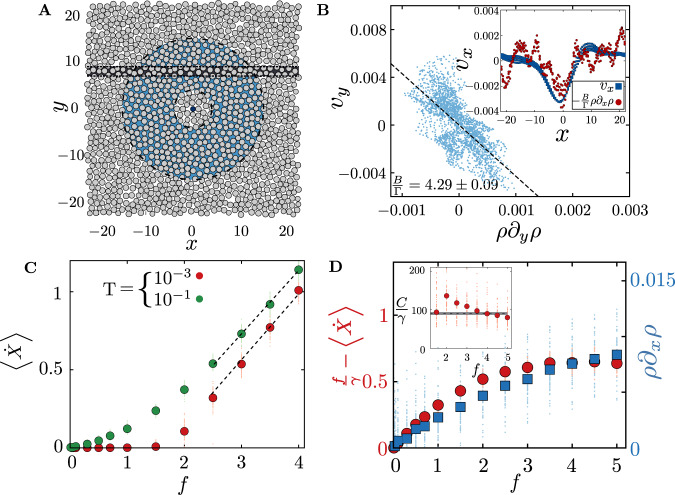
Verifying hydrodynamic equations by coarse-graining agent-based simulations. **A** Simulation snapshot of the *x*–*y* configurations showing an active particle at the origin (black dot) surrounded by particles comprising the compressible medium. We compute the coarse-grained fields, *ρ*(**r**, *t*), **v**(**r**, *t*) and their spatial derivatives in the shaded (blue) annular region and the thin shaded (black) rectangular region (Supplementary Note 3). **B** To verify (4), we plot *v*_*y*_(**r**, *t*) vs. *ρ*∂_*y*_*ρ* in the blue-shaded region in **A** at a given time; the best-fit line (dashed line) gives the parameter *B*/Γ = 4.29 ± 0.093. (inset) Using this value of *B*/Γ, we compute *v*_*x*_(**r**, *t*) and *ρ*∂_*x*_*ρ* vs. *x* along the thin black rectangular region in **A** and find good agreement. **C** Mean velocity of the active particle $\dot{X}$ (red and green dots) vs. active force, *f*, showing the dynamical transition between self-trapping at low *f* and movement. The mean is obtained by averaging over different initial realisations of the passive medium. Following (5), the value of *γ* can be extracted from the slope at large *f*. **D** To verify (5), we plot $f/\gamma -\dot{X}$ and *ρ*∂_*x*_*ρ* (just in front of the active particle) vs. *f*. This allows us to compute the parameter *C*/*γ* (inset), a measure of the back-reaction of the medium on the motile particle. The value of *C*/*γ* ≈ 150 is an order of magnitude larger than *B*/Γ and drops down to 100 at larger *f*. For **C** and **D** the smaller background symbols indicate data from individual simulations.

Take the case of a single particle, with no orientational fluctuations—Fig. 5A shows a simulation snapshot of an active particle surrounded by the compressible medium. We compute the coarse-grained fields, *ρ*(**r**, *t*), **v**(**r**, *t*) and their spatial derivatives—these results appear in Fig. 5B–D. To verify (4), we plot *v*_*y*_(**r**, *t*) vs. *ρ*∂_*y*_*ρ* to obtain *B*/Γ, from which we compute *v*_*x*_(**r**, *t*). The relation between the local velocity of the medium and the pile up of the density embodied in (4) is shown to hold up in Fig. 5B, even making allowance for a possible density dependent coefficient *B*/Γ (the contribution from viscous dissipation is significantly lower than the rest and so we drop it). We find that there is a dynamical transition between self-trapping at low *f* and movement (Fig. 5C). From this, we obtain the value of *γ* from the slope using (5) and hence *C*/*γ* (Fig. 5D). From this we see that the form of the back-reaction embodied in (5) is also borne out in Fig. 5D, albeit with a density (or force) dependent *C*/*γ* (inset Fig. 5D). Note that, consistent with our discussion above, *C*/*γ* is an order of magnitude larger than *B*/Γ. The fact that *C*/*γ* drops suddenly beyond *f* ≈ 2.5, would suggest that the friction experienced by the active particle increases with increasing *f* and then saturates to a constant value. In principle, if this drop is large enough, this could lead to an active discontinuous shear thickening^51^.

### Linearised hydrodynamics of a single active particle moving in a compressible medium

We first look at the dynamics of a single active particle in the compressible medium. Let us take the limit of large *τ*, and so over the timescale of interest, the orientation **n** is fixed, say along the $\hat{x}$ direction. As the active particle moves through the medium, it creates density inhomogeneities, which relax over time. Using (3) and (4) we get,

7 $${\partial }_{t}\rho+\frac{B}{{{\Gamma }}}\nabla \cdot \left({\rho }^{2}\nabla \rho \right)+\frac{f}{{{\Gamma }}}{\partial }_{x}\left(\rho {\delta }^{(2)}(x-X(t) ,\, y)\right)=0.$$

This nonlinear equation resembles an anisotropic Burgers equation with a source^52^, and so one might expect travelling pulse solutions. To see this explicitly, we perform a linear analysis about the uniform density of the ploughed medium. In this limit, we take **Γ** and *B* to be independent of *ρ*. Again in this limit, we ignore the back-reaction term *Cρ* ∇ *ρ* in (5); we will say that the active velocity, *v*_0_, is reduced from its bare value *f*/*γ* in a *ρ* dependent manner.

After initial transients, the density excess of the medium can then be written in terms of the collective coordinate,

$$\rho ({{{{{{{\bf{r}}}}}}}} ,\, t)=\rho ({{{{{{{\bf{r}}}}}}}}-{{{{{{{\bf{R}}}}}}}}(t))$$

Analytical solutions of the resulting linearised equation can be easily obtained by transforming the equation to coordinates in the moving frame of the active particle, *u* = *x* − *X*(*t*) and *w* = *y*, followed by Fourier transforming in (*u*, *w*) (see Supplementary Note 4). The excess density profile *ρ*(*u*, *w*) in the co-moving frame takes the form,

8 $$\rho (u ,\, w)=2{{{{{{{\mathcal{D}}}}}}}}(u)\left[{{{{{{{{\mathcal{I}}}}}}}}}_{1}(u ,\, w)+\frac{{{{{{{{{\mathcal{I}}}}}}}}}_{2}(u ,\, w)}{\xi }\right]$$

where,

$${{{{{{{{\mathcal{I}}}}}}}}}_{1}(u ,\, w)	=\frac{u/\xi }{\sqrt{{u}^{2}+{w}^{2}}}{K}_{1}\left[\frac{{\left({u}^{2}+{w}^{2}\right)}^{\frac{1}{2}}}{\xi }\right]\\ {{{{{{{{\mathcal{I}}}}}}}}}_{2}(u ,\, w)	={K}_{0}\left[\frac{{\left({u}^{2}+{w}^{2}\right)}^{\frac{1}{2}}}{\xi }\right]$$

with *K*_0_ and *K*_1_ being the modified Bessel functions of the second kind, and

9 $${{{{{{{\mathcal{D}}}}}}}}(u)=\frac{({\rho }_{0}+\rho (0 ,\, 0))f}{2\pi {v}_{0}\xi {{\Gamma }}}{{{{{{{{\rm{e}}}}}}}}}^{-\frac{u}{\xi }}$$

The decay length *ξ* is given by,

10 $$\xi \,\,=\frac{2B{\rho }_{0}^{2}}{{{\Gamma }}{v}_{0}}.$$

Since the fixed direction of motility breaks rotational invariance, it is natural to expect an anisotropy in the density profile. However, what comes as a surprise is that moving density profile breaks fore-aft symmetry. This is most apparent when we set *w* = 0^+^, and use the asymptotic expansion^53^

11 $${K}_{0}(z) \sim \sqrt{\frac{\pi }{2|z|}}{e}^{-z}\left(1-\frac{1}{8z}+\ldots \right)$$

and

12 $${K}_{1}(z) \sim \sqrt{\frac{\pi }{2|z|}}{e}^{-z}\left(1+\frac{3}{8z}+\ldots \right)$$

for large *z*. We see immediately that in the moving frame, the density profile in front to the motile particle is piled up and decays exponentially over a scale *ξ* from the pile up. The larger the active force *f*, the smaller is *ξ*, implying a sharper pile up. However, behind the active particle there is a long-range under-dense region, which decays as a power-law ∣*u*∣^−3/2^ (Supplementary Note 4).

**Fig. 6:**
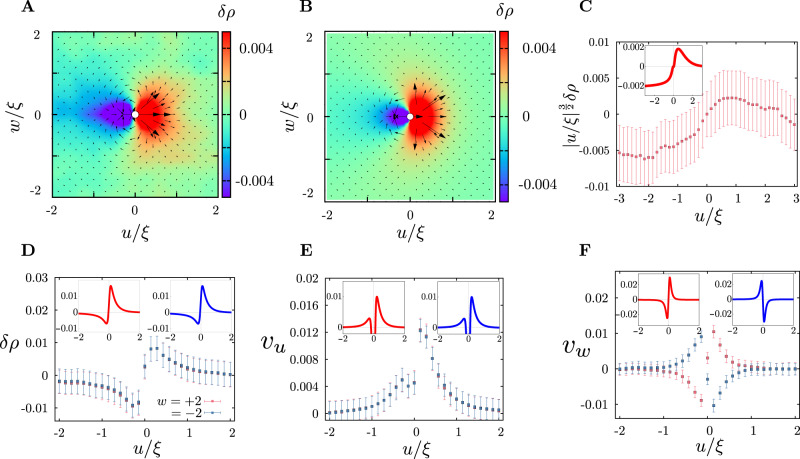
Density profile and flow field surrounding a single active particle moving through the passive medium. **A** Density profile (heat map) and velocity flow field (arrows, scaled for better visualisation) of the medium from simulations (in the co-moving frame (*u*, *w*) scaled by the decay length *ξ* ≈ 14) at *ϕ* = 0.45, *T* = 0.1, *f* = 2.0, and *τ* → *∞*. **B** Corresponding density profile (heat map) and velocity flow field (arrows, scaled for better visualisation) of the medium obtained from the linearised hydrodynamic theory. **C** The linearised theory (inset) predicts that the excess density of the passive medium is fore-aft asymmetric and shows an exponential decay in front and an algebraic decay behind the moving active particle, which is borne out by the simulations. The dark solid symbols show averages from multiple simulation runs, together with standard deviation. **D**–**F** The profiles of excess density and velocity components in the moving frame along *u* at *w*/*ξ* = 0^+^ (red) and *w*/*ξ* = 0^−^ (blue). Insets are the results from the linearised hydrodynamic equations. The dark solid symbols show averages from multiple simulation runs, together with standard deviation. The data presented here are averaged over time and 256 independent simulations and the errorbars, denoting the standard deviations, are estimated from this ensemble).

Knowing the density profile to linear order, we use (4) and (5) to compute the velocity flow field of the passive fluid and the velocity of the active particle. A comparison of the density profiles and the velocity flows with the simulation results is shown in Fig. 6A–F. The agreement is satisfying; in particular the demonstration in Fig. 6C that the excess density profile behind the moving active particle decays as the advertised power-law. One may, in principle, improve on the linear theory by setting up a diagrammatic perturbation expansion. However, since the linear theory compares well with the numerical simulation of (17) and with the “exact” numerical solution of the nonlinear equation (7) in *d* = 1 (next section), we do not take this up here.

### Accuracy of linear theory—comparison with “exact” numerical analysis of nonlinear equation in *d* = 1

A linear analysis in *d* = 1, shows that the density profile is,

13 $$\rho (u)	=\frac{({\rho }_{0}+\rho (0 ,\, 0))f}{{v}_{0}\xi {{\Gamma }}}{{{{{{{{\rm{e}}}}}}}}}^{-\frac{u}{\xi }}\quad \,{{\mbox{for}}}\,u\; > \; 0{{\mbox{}}}\\ 	=0\quad \,{{\mbox{for}}}\,u\; < \; 0{{\mbox{}}} , $$

where the length scale is given by $\xi=B{\rho }_{0}^{2}/{{\Gamma }}{v}_{0}$ (Supplementary Note 5). The density piles up in front of the active particle and decays exponentially ahead of it, while behind the active particle there is no wake. We now check to see how this calculated profile compares with an exact numerical solution of the nonlinear equation (7). The accurate numerical solution of this nonlinear PDE requires some care due to shock forming tendencies in the convective term (Supplementary Note 6). The result for the density profile of the travelling pulse is shown in Supplementary Fig. 5. The comparison with the linear theory is quite good, the absence of the wake is vividly apparent in the one-dimensional exact numerical solution (Supplementary Fig. 5).

### Two active particles moving through the compressible medium

The fore-aft asymmetric long-range density wake around the motile particle has an unusual effect on the interactions between two or more motile particles. This is best illustrated by considering the dynamics of two motile particles in a simplifying geometry where both particles move in the same direction with their separation vector being parallel or perpendicular to the direction of motion.

Let the trajectories of the two active particles be represented by **R**_1_(*t*), **R**_2_(*t*), in the limit of large persistence time, so that we can take the orientations **n**_1_ and **n**_2_ to be time independent. To ${{{{{{{\mathcal{O}}}}}}}}(\delta \rho )$, these particles, individually, leave a time dependent anisotropic and fore-aft asymmetric wake described by *ρ*(*x*, *y*, *t*), whose back-reaction on the movement of the active particles themselves, is easily estimated

14 $$\gamma {\dot{{{{{{{{\bf{R}}}}}}}}}}_{1}=f{{{{{{{{\bf{n}}}}}}}}}_{1}-C{\rho }_{0}\nabla \rho {\left|\right.}_{{{{\mbox{self}}}}_{1}}-C{\rho }_{0}\nabla \rho {\left|\right.}_{1\leftarrow 2}$$

15 $$\gamma {\dot{{{{{{{{\bf{R}}}}}}}}}}_{2}=f{{{{{{{{\bf{n}}}}}}}}}_{2}-C{\rho }_{0}\nabla \rho {\left|\right.}_{{{{\mbox{self}}}}_{2}}-C{\rho }_{0}\nabla \rho {\left|\right.}_{2\leftarrow 1}$$

where *i* ← *j* denotes the effect of particle *j* on particle *i*. These equations may be cast in terms of the relative coordinate **R**_*rel*_ = **R**_1_ − **R**_2_ and the centre of mass **R**_*cm*_ = (**R**_1_ + **R**_2_)/2. For the two geometries under consideration, we find the following (details in Supplementary Note 4).

**Fig. 7:**
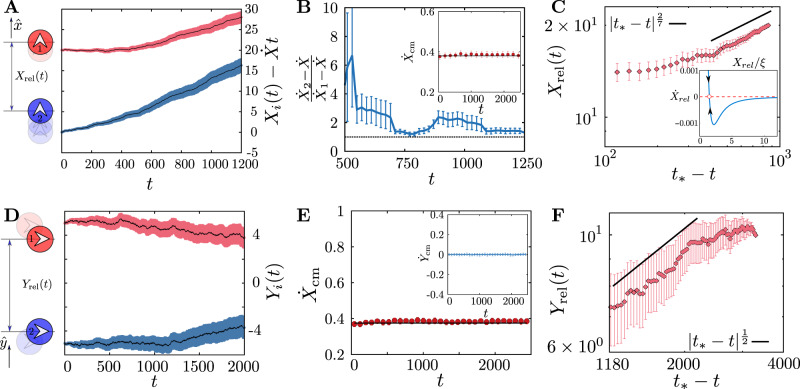
Dynamics of two particles moving through the passive medium. **A**–**C**. Two active particles moving along the *x*-axis (the direction of their active forces whose magnitude *f* = 2, and large persistence time *τ*) with separation vectors parallel to the direction of motion. **A** Time dependence of the positions of the pair of active particles (after subtracting the measured single particle displacement $\dot{X}t$), along $\hat{x}$. **B** The ratio of the particle velocities remains greater than one, indicating a speed up of the trailing particle towards the leading particle, a manifestation of *nonreciprocity*. Inset: the centre of mass velocity ${\dot{X}}_{{{{{{{{\rm{cm}}}}}}}}}(t)$ remains positive and constant (solid line is the prediction from theory). **C** The two active particles approach each other in finite time t_*_. The solid line, a prediction from theory, suggests a scaling form *X*_rel_ ∝ ∣*t* − t_*_∣^*α*^, where *α* = 2/7. As the second particle approaches the first, it forms a bound state with the first particle, characterised by the existence of a stable fixed point at *X*_rel_ ≈ 1.5064*ξ* (inset), verified in the the numerical simulations as a flattening of *X*_rel_ as *t* approaches t_*_. **D**–**F** Two active particles moving along the *x*-axis with separation vectors perpendicular to the direction of motion (rest same as above). **D** Time dependence of *y*-positions of the active particles showing convergent trajectories. **E** The x-component of centre of mass velocity is positive and constant, solid line is the prediction from theory. Inset shows y-component centre of mass velocity is zero, indicating symmetric approach. **F** The two active particles approach each other in finite time t_*_. The solid line, a prediction from theory, suggests that the relative position has a scaling form *Y*_*rel*_ ∝ ∣*t* − t_*_∣^*α*^, where *α* = 1/2 as *t* → t_*_. The data presented here are averaged over 256 independent simulations and the errorbars, denoting the standard deviations, are estimated from this ensemble.

1. The separation vector between the leading particle 1 and the trailing particle 2 is along the $\hat{x}$ direction, parallel to their direction of motion (Fig. 7A).
  We find that while the centre of mass velocity ${\dot{X}}_{cm} \, > \, 0$ (Fig. 7B inset), the inter-particle separation *X*_rel_ decreases in time (Fig. 7A), i.e., ${\dot{X}}_{{{{{{\mathrm{rel}}}}}}} \, < \, 0$, starting from the initial value, till it reaches *X*_rel_ ≈ 1.5064*ξ*, which is a stable fixed point of the dynamics. This corresponds to a bound state of the two particles in this linearised theory, which appears to be consistent with the simulations (Fig. 7C). The speed of approach of the particle 2 to particle 1 first increases slowly and then rapidly decreases to zero as the bound state is reached (Fig. 7C, inset). This nonreciprocal sensing^2,3,6^, is a consequence of the fore-aft asymmetric wake and causes the trailing particle to catch up with the leading one in finite time (Fig. 7B).
  A dominant balance analysis of the equation for *X*_rel_ shows an early time scaling of the form *X*_rel_ ∝ ∣*t* − t_*_∣^2/7^ as the particles approach each other (Fig. 7C).
2. The separation vector between the particle 1 and the particle 2 is along the $\hat{y}$ direction, perpendicular to their direction of motion (Fig. 7D).
  Here again the centre of mass velocity ${\dot{X}}_{{{{{{\mathrm{cm}}}}}}} \, > \, 0$ (and is the same as the single particle speed, Fig. 7E), and the inter-particle separation *Y*_rel_ decreases in time (Fig. 7D), i.e., ${\dot{Y}}_{{{{{{\mathrm{rel}}}}}}} \, < \, 0$, starting from the initial value. This leads to the trajectories of particles 1 and 2 converging towards each other in a symmetrical manner (Fig. 7D).
  An asymptotic analysis shows that the relative position *Y*_rel_ ∝ ∣*t* − t_*_∣^1/2^ as *t* → t_*_ (Fig. 7F).

The second case resembles the magnetic force between two parallel wires carrying current in the same direction, and is a consequence of the breaking of time reversal symmetry. Likewise, a pair of active particles initially moving towards each other, will scatter off (repel) and will slow down as they move away from each other. The scattering of active particles in other geometries can also be worked out to this order.

## Discussion

In this paper, we have studied the dynamics of a dilute suspension of active Brownian particles moving through a dense compressible passive fluid that dissipates momentum through friction. The dynamical interplay between the active particles and the passive medium, not only results in a remodelling of the passive medium, but also in a back-reaction on the movement of the active particles themselves. Such active *ploughers* show a jamming transition at fixed density of the medium. In the unjammed phase, a moving active plougher generates a fore-aft asymmetric density wake, which is the source of the long-range nonreciprocal interaction between moving active particles mediated dynamically through the passive compressible medium. This emergent nonreciprocal interaction is a consequence of a dynamical phase transition to a state with finite current. This leads to a nonreciprocal sensing wherein a trailing particle senses and catches up with a leading particle moving ahead of it. Further, the movement of the active particle leaves a dynamical trace on the responsive medium, these effects of nonreciprocity are more indelibly manifest in the vicinity of the jamming-unjamming transition.

We recall that in (6) we have assumed that the direction of the propulsion force is set by some mechanism internal to the active particle and therefore independent of the passive medium. To experience the full scope of nonreciprocal effects possible here, one needs to extend the hydrodynamic equations (5), (6), to include an active torque that drives **n**_*i*_ to align along the direction of the smallest (largest) density gradient ∇ *ρ*—this will lead to both *taxis* and *phoresis*, features that have been explored in refs. 3, 6 in other contexts. Such considerations lead to a simple physical version of sense-and-capture, even in the absence of any kind of chemical sensing.

The success of our hydrodynamic analysis motivates us to go beyond the study of one and two active particles and look at many-body effects^54^. In ref. 15, we had seen how the minority component self-propelled particles cluster on account of activity; the long-range nonreciprocal interaction observed here, will translate to a new kind of *nonreciprocal motility induced clustering*^42^ of active particles mediated by the passive medium. This and its relationship with the anisotropic Burgers equation with coloured noise^52^ will be taken up later.

## Methods

### Agent-based simulations

We work with a modified binary mixture (see Supplementary Note 1 for details), at a fixed area fraction, *ϕ*, where particles interact via a potential,

16 $${V}_{ij}=4{\epsilon }_{ij}{\left(\frac{{\sigma }_{ij}}{{r}_{ij}}\right)}^{12}+{v}_{0}+{v}_{2}{\left(\frac{{\sigma }_{ij}}{{r}_{ij}}\right)}^{-2}+{v}_{4}{\left(\frac{{\sigma }_{ij}}{{r}_{ij}}\right)}^{-4}$$

if *r*_*ij*_ < *r*_*c*,*ij*_ or 0 otherwise. We fix the energy and length scales to be in the units of *ϵ*_AA_ and *σ*_AA_, respectively.

Of these a small fraction of particles *ϕ*_*a*_ is made active—their dynamics is described by active Brownian particles (ABP) (see Supplementary Note 1 for details) immersed in a background of passive particles. All particles are subject to a thermal noise *ϑ* of zero mean and variance equal to 2*γT* (setting *k*_B_ = 1), obeying FDT. The subset $i\in {{{{{{{\mathcal{A}}}}}}}}$ of ABPs are subject to additional active stochastic forces ${{{{{{{{\bf{f}}}}}}}}}_{i}=f\,{{{{{{{{\bf{n}}}}}}}}}_{i}\equiv f\,\left(\cos {\theta }_{i} ,\,\sin {\theta }_{i}\right)$. The orientation of the propulsion force *θ*_*i*_ undergoes rotational diffusion, described by an athermal noise *ξ*_*i*_, with zero mean and correlation $\langle {\xi }_{i}(t){\xi }_{j}(t^{\prime} )\rangle=2{\tau }^{-1}{\delta }_{ij}\delta (t-t^{\prime} )$.

The full dynamics is described by the Langevin equation,

17 $$m{\ddot{{{{{{{{\bf{x}}}}}}}}}}_{i}	=-\gamma {\dot{{{{{{{{\bf{x}}}}}}}}}}_{i}-{\partial }_{i}\mathop{\sum }\limits_{j\ne i}^{N}{V}_{ij}+f{{{{{{{{\bf{n}}}}}}}}}_{i}\,{{\mathbb{1}}}_{(i\in {{{{{{{\mathcal{A}}}}}}}})}+{{{\mathbf{\vartheta}}}}_{i} , \\ {\dot{\theta }}_{i}	={\xi }_{i}\quad {{{{{{{\rm{for}}}}}}}}\,i\in {{{{{{{\mathcal{A}}}}}}}}.$$

where ${{\mathbb{1}}}_{(i\in {{{{{{{\mathcal{A}}}}}}}})}$ is the indicator function, which ensures that the active forces are only imposed on particles *i* belonging to the active set ${{{{{{{\mathcal{A}}}}}}}}$.

We perform Brownian dynamics (BD) simulations at fixed particle-number, volume of the system and temperature of the heat-bath (NVT) in 2-dimensions using a square box of reduced length *L*_0_ = 45 and 90, with periodic boundary conditions (PBC). For all the simulations related to Figs. 1 and 2, we keep the area fraction of active particles fixed at *ϕ*_*a*_ = 0.017 (dilute limit), and vary the number of passive particles constituting the medium to control the overall density or area fraction *ϕ*. The simulations to generate data for Figs. 3, 4, 5, 6 are performed with one active particle in the passive medium.

### Numerical solution of nonlinear hydrodynamic equations in *d* = 1

A finite volume discretization with an exponential scheme for the convective flux was used to numerically solve the 1-dimensional nonlinear hydrodynamic equations (see Supplementary Note 6). The flux at the interface of the *i*^th^ and (*i*−1)^th^ grid in this scheme is given by

18 $${J}_{x}	=\frac{D}{{{\Delta }}x}\left(B{\rho }^{i-1}-A{\rho }^{i}\right)\\ A	=\frac{{{{{{\mathrm{Pe}}}}}}}{{e}^{{{{{{\mathrm{Pe}}}}}}}-1}\\ B	=A+{{{{{\mathrm{Pe}}}}}} , $$

where ${{{{{\mathrm{Pe}}}}}}=\frac{{{{{{{{\bf{v}}}}}}}}{{\Delta }}x}{D}$ is the Péclet number and Δ*x* is the grid spacing. This scheme guarantees positive solutions and has low diffusive error as the flux is formulated using the exact solution. A first order temporal discretization was used in combination with a sweep between each iteration using Newton’s method. We used the PDE solver available in the NIST-FiPy package to obtain the numerical solutions.

## Supplementary information

Supplementary Information

## Data Availability

No additional data was used besides the results of numerical simulations using the parameters described in the text. Additional summary statistics of the data plotted may be available upon reasonable request.

## Code availability

All code used to produce these results is available at https://github.com/jyotiprasad/UnjammingEmergentNonreciprocity.

## References

[1] Vishen AS, Prost J, Rao M (2019). Breakdown of effective temperature, power law interactions and self-propulsion in a momentum conserving active fluid. Phys. Rev. E.

[2] Gupta RK, Kant R, Soni H, Sood AK, Ramaswamy S (2022). Active nonreciprocal attraction between motile particles in an elastic medium. Phys. Rev. E.

[3] Saha S, Golestanian R, Ramaswamy S (2014). Clusters, asters, and collective oscillations in chemotactic colloids. Phys. Rev. E.

[4] Ramaswamy S, Simha RA, Toner J (2003). Active nematics on a substrate: Giant number fluctuations and long-time tails. Europhys. Lett..

[5] Marchetti MC (2013). Hydrodynamics of soft active matter. Rev. Mod. Phys..

[6] Husain K, Rao M (2017). Emergent structures in an active polar fluid: dynamics of shape, scattering, and merger. Phys. Rev. Lett..

[7] Saha S, Agudo-Canalejo J, Golestanian R (2020). Scalar active mixtures: the nonreciprocal Cahn-Hilliard model. Phys. Rev. X.

[8] You Z, Baskaran A, Cristina Marchetti M (2020). Nonreciprocity as a generic route to traveling states. Proc. Natl Acad. Sci. USA.

[9] Fruchart M, Hanai R, Littlewood PB, Vitelli V (2021). Non-reciprocal phase transitions. Nature.

[10] Dzubiella J, Löwen H, Likos CN (2003). Depletion forces in nonequilibrium. Phys. Rev. Lett..

[11] Das J, Rao M, Ramaswamy S (2002). Driven Heisenberg magnets: nonequilibrium criticality, spatiotemporal chaos and control. Europhys. Lett..

[12] Das, J, Rao, M. & S. Ramaswamy, S. Nonequilibrium steady states of the isotropic classical magnet. Preprint at https://arxiv.org/pdf/cond-mat/0404071.pdf (2004).

[13] Reichhardt C, Reichhardt CJO (2015). Active microrheology in active matter systems: Mobility, intermittency, and avalanches. Phys. Rev. E.

[14] Bechinger C (2016). Active particles in complex and crowded environments. Rev. Mod. Phys..

[15] Mandal R, Bhuyan PJ, Rao M, Dasgupta C (2016). Active fluidization in dense glassy systems. Soft Matter.

[16] Mandal R, Bhuyan PJ, Chaudhuri P, Dasgupta C, Rao M (2020). Extreme active matter at high densities. Nat. Commun..

[17] Henkes S, Fily Y, Marchetti MC (2011). Active jamming: Self-propelled soft particles at high density. Phys. Rev. E.

[18] Ni R, CohenStuart MA, Dijkstra M (2013). Pushing the glass transition towards random close packing using self-propelled hard spheres. Nat. Commun..

[19] Berthier L, Kurchan J (2013). Non-equilibrium glass transitions in driven and active matter. Nat. Phys..

[20] Berthier L (2014). Nonequilibrium glassy dynamics of self-propelled hard disks. Phys. Rev. Lett..

[21] Berthier L, Flenner E, Szamel G (2019). Glassy dynamics in dense systems of active particles. J. Chem. Phys..

[22] Parry BR (2014). The bacterial cytoplasm has glass-like properties and is fluidized by metabolic activity. Cell.

[23] Nishizawa K (2017). Universal glass-forming behavior of in vitro and living cytoplasm. Sci. Rep..

[24] Hameed FM, Rao M, Shivashankar GV (2012). Dynamics of passive and active particles in the cell nucleus. PLoS ONE.

[25] Bhattacharjee T, Datta SS (2019). Bacterial hopping and trapping in porous media. Nat. Commun..

[26] Bhattacharjee T, Datta SS (2019). Confinement and activity regulate bacterial motion in porous media. Soft Matter.

[27] Angelini TE (2011). Glass-like dynamics of collective cell migration. Proc. Natl Acad. Sci. USA.

[28] Jiang C, Cui C, Li L, Shao Y (2014). The anomalous diffusion of a tumor invading with different surrounding tissues. PLoS ONE.

[29] Malmi-Kakkada AN, Li X, Samanta HS, Sinha S, Thirumalai D (2018). Cell growth rate dictates the onset of glass to fluid-like transition and long time superdiffusion in an evolving cell colony. Phys. Rev. X.

[30] Gravish N, Monaenkova D, Goodisman MAD, Goldman DI (2013). Climbing, falling, and jamming during ant locomotion in confined environments. Proc. Natl Acad. Sci. USA.

[31] Espinoza DN, Santamarina JC (2010). Ant tunneling-a granular media perspective. Granul. Matter.

[32] de Macedo RB (2021). Unearthing real-time 3D ant tunneling mechanics. Proc. Natl Acad. Sci. USA.

[33] Capowiez Y, Bottinelli N, Sammartino S, Michel E, Jouquet P (2015). Morphological and functional characterisation of the burrow systems of six earthworm species (Lumbricidae). Biol. Fertil. Soils.

[34] Lozano C, Gomez-Solano JR, Bechinger C (2019). Active particles sense micromechanical properties of glasses. Nat. Mater..

[35] Singh K, Yadav A, Dwivedi P, Mangal R (2022). Interaction of active Janus particles with passive tracers. Langmuir.

[36] Vasilyev OA, Bénichou O, Mejía-Monasterio C, Weeks ER, Oshanin G (2017). Cooperative behavior of biased probes in crowded interacting systems. Soft Matter.

[37] Kob W, Andersen HC (1995). Testing mode-coupling theory for a supercooled binary Lennard-Jones mixture I: The van Hove correlation function. Phys. Rev. E.

[38] Brüning R, St-Onge DA, Patterson S, Kob W (2008). Glass transitions in one-, two-, three-, and four-dimensional binary Lennard-Jones systems. J. Phys. Condens. Matter.

[39] Fily Y, Marchetti MC (2012). Athermal phase separation of self-propelled particles with no alignment. Phys. Rev. Lett..

[40] Takatori SC, Brady JF (2015). Towards a thermodynamics of active matter. Phys. Rev. E.

[41] Levis D, Codina J, Pagonabarraga I (2017). Active Brownian equation of state: metastability and phase coexistence. Soft Matter.

[42] Cates ME, Tailleur J (2015). Motility-induced phase separation. Annu. Rev. Condens. Matter Phys..

[43] Mandal R, Sollich P (2020). Multiple types of aging in active glasses. Phys. Rev. Lett..

[44] Berthier L, Biroli G (2011). Theoretical perspective on the glass transition and amorphous materials. Rev. Mod. Phys..

[45] Gnan N, Das G, Sperl M, Sciortino F, Zaccarelli E (2014). Multiple glass singularities and isodynamics in a core-softened model for glass-forming systems. Phys. Rev. Lett..

[46] Zaccarelli E, Poon WCK (2009). Colloidal glasses and gels: the interplay of bonding and caging. Proc. Natl Acad. Sci. USA.

[47] Mason TG (2000). Estimating the viscoelastic moduli of complex liquids using the generalized Stokes-Einstein equation. Rheologica Acta.

[48] Tanaka H, Lee AA, Brenner MP (2017). Hot particles attract in a cold bath. Phys. Rev. Fluids.

[49] Aragones JuanL, Yazdi S, Alexander-Katz A (2018). Diffusion of self-propelled particles in complex media. Phys. Rev. Fluids.

[50] Hokmabad BV, Agudo-Canalejo J, Saha S, Golestanian R, Maass CC (2022). Chemotactic self-caging in active emulsions. Proc. Natl. Acad. Sci. USA.

[51] Wyart M, Cates ME (2014). Discontinuous shear thickening without inertia in dense non-Brownian suspensions. Phys. Rev. Lett..

[52] Medina E, Hwa T, Kardar M, Zhang YC (1989). Burgers equation with correlated noise: renormalization-group analysis and applications to directed polymers and interface growth. Phys. Rev. A.

[53] Abramowitz, M., Stegun, I. A. & Romer, R. H. *Handbook of Mathematical Functions with Formulas, Graphs, and Mathematical Tables* (American Association of Physics Teachers, 1988).

[54] Reichhardt C, Reichhardt CJO (2006). Cooperative behavior and pattern formation in mixtures of driven and nondriven colloidal assemblies. Phys. Rev. E.

