# Supplementary material for: Unjamming and emergent nonreciprocity in active ploughing through a compressible viscoelastic fluid
Source: Nat Commun. 2022 Aug 4;13:4533. doi: 10.1038/s41467-022-31984-z (PMC9352703; doi:10.1038/s41467-022-31984-z)
Supplement: Supplementary file 1 — Supplementary Information [file 41467_2022_31984_MOESM1_ESM.pdf]

Supplementary Information

Unjamming and emergent nonreciprocity in active ploughing through a compressible viscoelastic fluid

Jyoti Prasad Banerjee,[1,*] Rituparno Mandal,[2,*] Deb Sankar Banerjee,[3] Shashi Thutupalli,[1,4,†] and Madan Rao[1,‡]

[1] *Simons Centre for the Study of Living Machines, National Centre for Biological Sciences (TIFR), Bangalore, India*

[2] *Institute for Theoretical Physics, Georg-August-Universität Göttingen, 37077 Göttingen, Germany*

[3] *Department of Physics, Carnegie Mellon University, Pittsburgh, USA*

[4] *International Centre for Theoretical Sciences (TIFR), Bangalore, India*

CONTENTS

Supplementary Note 1. Agent-based simulations S3

Supplementary Note 2. Characterisation of approach to glass transition S4

Supplementary Note 3. Morphology of underdense regions in the medium excavated by the active particles S5
A. Creation of a density map from agent-based simulations S5
B. Identification of under-dense regions and analysis of their geometry S6

Supplementary Note 4. Linearised hydrodynamic theory S6
A. Single active particle moving through the compressible medium in $d = 2$ S6
B. Two active particles moving through the compressible medium in $d = 2$ S10

Supplementary Note 5. Linearised hydrodynamics theory in $d = 1$: density profiles S13

Supplementary Note 6. Numerical solution of nonlinear hydrodynamic equations in $d = 1$ S14

Supplementary Note 7. Finite-size effects S14

References S15

Supplementary Note 1. AGENT-BASED SIMULATIONS

To ensure that the assembly of soft particles of area fraction ϕ remains in a positionally disordered state, we work with a modified 2D Kob-Andersen 65 : 35 (A:B) binary mixture [1, 2], a prototype glass-forming liquid. Particles i and j, interact via a 2-body soft repulsive potential,

$$V_{ij} = \begin{cases} 4\epsilon_{ij}\left(\frac{\sigma_{ij}}{r_{ij}}\right)^{12} + v_0 + v_2\left(\frac{\sigma_{ij}}{r_{ij}}\right)^{-2} + v_4\left(\frac{\sigma_{ij}}{r_{ij}}\right)^{-4}, & \text{if } r_{ij} < r_{c,ij} \\ 0, & \text{otherwise} \end{cases} \tag{S1}$$

where r_{ij} is the distance between the particles i and j. Our choice of parameter values, $v_0 = -112\epsilon_{ij}$, $v_2 = 192\epsilon_{ij}$, $v_4 = -84\epsilon_{ij}$ and the cut-off $r_{c,ij} = \sigma_{ij}$, ensures that both the potential and the force is smooth at the cut-off distance and the interaction is purely repulsive. We fix the energy and length scales to be in the units of ϵ_{AA} and σ_{AA}, respectively by setting $\epsilon_{AA} = 1.0$, $\sigma_{AA} = 1.0$. The other parameters are chosen following the Kob-Andersen model [1] *i.e.* $\epsilon_{BB} = 0.50$, $\sigma_{BB} = 0.88$, $\epsilon_{AB} = 1.50$, $\sigma_{AB} = 0.80$.

Of these a small fraction of particles ϕ_a is made active - their dynamics is described by active Brownian particles (ABP) [3–8] immersed in a background of passive particles. All particles are subject to a thermal noise ϑ of zero mean and variance equal to $2\gamma T$ (setting $k_B = 1$), obeying FDT. The subset $i \in \mathcal{A}$ of ABPs are subject to additional active stochastic forces $\mathbf{f}_i = f\,\mathbf{n}_i \equiv f\,(\cos\theta_i, \sin\theta_i)$. The orientation of the propulsion force θ_i undergoes rotational diffusion, described by an athermal noise ξ_i, with zero mean and correlation $\langle \xi_i(t)\xi_j(t')\rangle = 2\tau^{-1}\delta_{ij}\delta(t-t')$. Its effect on the particle-dynamics appears as an exponentially correlated vectorial noise with correlation time τ, which being unrelated to the drag γ, is not constrained by fluctuation-dissipation relation.

The full dynamics is described by the Langevin equation,

$$\begin{aligned} m\ddot{\mathbf{x}}_i &= -\gamma\dot{\mathbf{x}}_i - \partial_i \sum_{j\neq i}^{N} V_{ij} + f\mathbf{n}_i\,\mathbb{1}_{(i\in\mathcal{A})} + \vartheta_i\,, \\ \dot{\theta}_i &= \xi_i \qquad \text{for } i \in \mathcal{A}\,. \end{aligned} \tag{S2}$$

where $\mathbb{1}_{(i\in\mathcal{A})}$ is the indicator function which ensures that the active forces are only imposed on particles i belonging to the active set $\mathcal{A}$.

To proceed with the numerical simulation, we take the overdamped limit (drop inertia) and convert the resulting dynamical equation to non-dimensionless form by scaling,

$$t \to \frac{\gamma}{\epsilon_{AA}}t,\;\; \mathbf{x}_i \to \sigma_{AA}\mathbf{x}_i,\;\; V_{ij} \to \epsilon_{AA}V_{ij},\;\; T \to \epsilon_{AA}T \tag{S3}$$

and setting $\sigma_{AA} = \epsilon_{AA} = 1$, to arrive at,

$$\frac{dx_i}{dt} = -\partial_i \sum_{j\neq i}^{N} V_{ij} + f(\mathbf{n}_i.\hat{\mathbf{x}}_i)\,\mathbb{1}_{(i\in\mathcal{A})} + \sqrt{\frac{2T}{dt}}\zeta_i \tag{S4}$$

where $\langle \zeta_i(t)\rangle = 0$ and $\langle \zeta_i(t)\zeta_j(t')\rangle = \delta_{ij}\delta(t-t')$. Similarly, the orientational dynamics (which is purely diffusive), can be written as,

$$\dot{\theta}_i = \sqrt{\frac{2}{\tau}}\eta_i \tag{S5}$$

where, $\langle \eta_i(t)\rangle = 0$ and $\langle \eta_i(t)\eta_j(t')\rangle = \delta_{ij}\delta(t-t')$.

We perform Brownian dynamics (BD) simulations at fixed particle-number, volume of the system and temperature of the heat-bath (NVT) in 2-dimensions using a square box of reduced length $L_0 = 45$ and 90, with periodic boundary conditions (PBC). We use predictor-corrector algorithm (Euler-Trapezoidal method) and forward Euler method to update the positions and orientations, respectively [9, 10]. For all the simulations related to Figs. 1, 2 (main text) we keep the area fraction of active particles fixed at $\phi_a = 0.017$ (dilute limit), and vary the number of passive particles constituting the medium to control the overall density or area fraction ϕ. The simulations to generate data for Figs. 3, 4, 5, 6 (main text) are performed with one active particle in the passive medium.

Supplementary Note 2. CHARACTERISATION OF APPROACH TO GLASS TRANSITION

For different values of the area fraction ϕ, we first equilibrate the passive medium at the reduced temperature $T = 0.5$. We then monitor the approach of the passive medium towards a glass transition, by computing the relaxation of its density fluctuations, measured by the two point overlap correlation function (Fig. Supplementary Figure 1**A**); the transport of tagged particles, measured by the mean squared displacement (Fig. Supplementary Figure 1**C**) and its rheological properties, as we systematically vary ϕ. Only in this section, τ indicates the time elapsed starting from the time-origin, t, and is not to be confused with the τ used in all the other sections of this *Supplementary Information* as well as in the main text, representing the persistence time of the active particles.

Overlap function, $Q(\tau)$, a measure of the density relaxation, is defined as

$$Q(\tau;\phi,T) = \left\langle \frac{1}{N}\sum_{i=1}^{N} \omega\left(|\mathbf{x}_i(t+\tau) - \mathbf{x}_i(t)|\right) \right\rangle \tag{S6}$$

with $\omega(x) = 1$ for $x < 0.3$ and zero otherwise. The $\langle ... \rangle$ sign indicates averaging over different time-origins (t), and different realisations of the system. The α-relaxation time, τ_α is calculated by setting

$$Q(\tau = \tau_\alpha;\phi,T) = \frac{1}{e} \tag{S7}$$

Then τ_α, for different area fractions ϕ, is fit to the Vogel-Fulcher–Tammann (VFT) form (Fig. Supplementary Figure 1**B**),

$$\tau_\alpha = \tau_\infty \exp\left(\frac{B}{\phi_{\mathrm{VFT}} - \phi}\right) \tag{S8}$$

to obtain ϕ_{VFT}, the VFT glass transition area fraction.

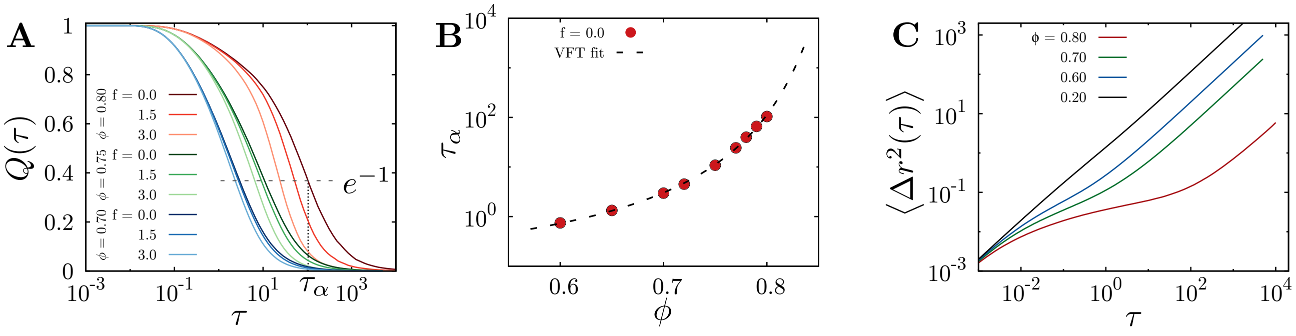

FIG. Supplementary Figure 1. **A** Q(τ) for three different area fractions of the medium and three different values of active force at $T = 0.5$. The gray dashed line represents $Q(\tau = \tau_\alpha, \phi, T) = e^{-1}$ as in (S7). The corresponding τ is τ_α, as shown by the black dotted line. **B** VFT fit of τ_α vs. area fraction (ϕ) for the passive medium ($f = 0.0$). **C** Mean squared displacements of the medium at different area fractions. The lowest area fraction ($\phi = 0.20$) represents the weak interaction limit, while the highest one ($\phi = 0.80$) shows stong caging effect upto $\tau \approx 10^2$.

Mean squared displacement (MSD), is defined as,

$$\left\langle \Delta r^2(\tau) \right\rangle = \left\langle \frac{1}{N}\sum_{i=1}^{N} |\mathbf{x}_i(t+\tau) - \mathbf{x}_i(t)|^2 \right\rangle \tag{S9}$$

The $\langle \dots \rangle$ denotes averaging over the time-origins and multiple statistically independent realizations. The long time diffusion coefficient (D_∞) is computed from the long time limit of the MSD,

$$D_\infty = \frac{1}{4\tau} \lim_{\tau\to\infty} \left\langle \Delta r^2(\tau) \right\rangle \tag{S10}$$

Passive microrheology, is a measure of the viscoelastic(shear) properties of the medium, and can be obtained from the frequency transformed MSD, $\langle \Delta r^2(\omega) \rangle$ [11],

$$G'(\omega) = |G^*(\omega)| \cos\left(\frac{\pi\alpha(\omega)}{2}\right)$$
$$G''(\omega) = |G^*(\omega)| \sin\left(\frac{\pi\alpha(\omega)}{2}\right) \tag{S11}$$

where,

$$|G^*(\omega)| \approx \frac{K_B T}{\pi a \langle \Delta r^2(\omega) \rangle \Gamma\left(1+\alpha(\omega)\right)} \tag{S12}$$

$$\alpha(\omega) = \left.\frac{d \ln \langle \Delta r^2(\tau) \rangle}{d \ln \tau}\right|_{\tau=\frac{1}{\omega}} \tag{S13}$$

Here, 'a' is the radius of the particle and $\Gamma(x)$ is the Γ-function.

We choose $T = 0.5$ for all the simulations related to Fig. 1 as (1) at this temperature cage-hopping dynamics is clearly visible for higher area fractions (i.e., $\phi = 0.7 - 0.8$) of the medium, and (2) for moderate area fractions (i.e., $\phi = 0.4 - 0.6$) the temperature is not too close to the glass transition temperature where the dynamics becomes extremely slow and it becomes very difficult to acquire enough data within a reasonable run-time. Both the equilibration and data-generation runs are for a time $\geq 100\ \tau_\alpha$.

Supplementary Note 3. MORPHOLOGY OF UNDERDENSE REGIONS IN THE MEDIUM EXCAVATED BY THE ACTIVE PARTICLES

A. Creation of a density map from agent-based simulations

We use the instantaneous configuration (i.e., position-coordinates of all the passive particles) to generate a coarse-grained density field that has been used in Figs. 3-5 in the main text. For this, we divide the entire simulation box of size L_0 into a square grid of grid length L_g, and number of cells $N_g = (\frac{L_0}{L_g})^2$. For a typical cell with indices (k, l), we compute the density field using the following formulae

$$\rho(k, l) = \frac{1}{\mathcal{N}} \sum_{i=1}^{N} \exp\left(-\frac{d}{d_0}\right) \tag{S14}$$

where $d = |\mathbf{x}_i - \mathbf{x}_{k,l}|$ is the distance of the i-th particle from the centre of the grid ($\mathbf{x}_{k,l}$) and d_0 is the coarse-graining length scale, and $\mathcal{N}$ is the normalisation constant. We have used different values for L_g and d_0, ensuring that $d_0 > L_g$ and that both are $\sim \mathcal{O}(1)$. For Fig. 3, 4 since we compare the density fields between different area fractions, we choose

$$\mathcal{N} = \sum_{k,l=1}^{\sqrt{N_g}} \sum_{i=1}^{N} \exp\left(-\frac{d}{d_0}\right) \tag{S15}$$

which ensures that (S14) gives us the normalised probability density of the passive medium. For Fig. 5 and Fig. 6 (main text), we choose

$$\mathcal{N} = \frac{1}{N} \sum_{k,l=1}^{\sqrt{N_g}} \sum_{i=1}^{N} \exp\left(-\frac{d}{d_0}\right) \tag{S16}$$

which ensures that (S14) gives us normalised number density.

B. Identification of under-dense regions and analysis of their geometry

We use the density profile that we discussed in Sect. Supplementary Note 3 A and identify the low density regions by employing an upper cut-off ($\sim 4 \times 10^{-4}$) on the grid defined above. The results described in Fig. 4**A** in main text are robust to reasonable variation of this threshold. Any grid point (cell) that has density less than or equal to the aforementioned threshold is considered as low density region. This low density regions are represented by a set of points defined on the grid $\mathbf{z}_i$, where $i \in [1, N_u]$ where N_u is the total number of under-dense grid points. We then compute moment of gyration tensor, $M_{\alpha\beta} = \sum_{i=1}^{N_u}(z_i^\alpha - z_{\rm cm}^\alpha)(z_i^\beta - z_{\rm cm}^\beta)$, to characterise the spatial distribution or structures of such point where $\mathbf{z}_{\rm cm} = \frac{1}{N_u}\sum_{i=1}^{N_u}\mathbf{z}_i$.

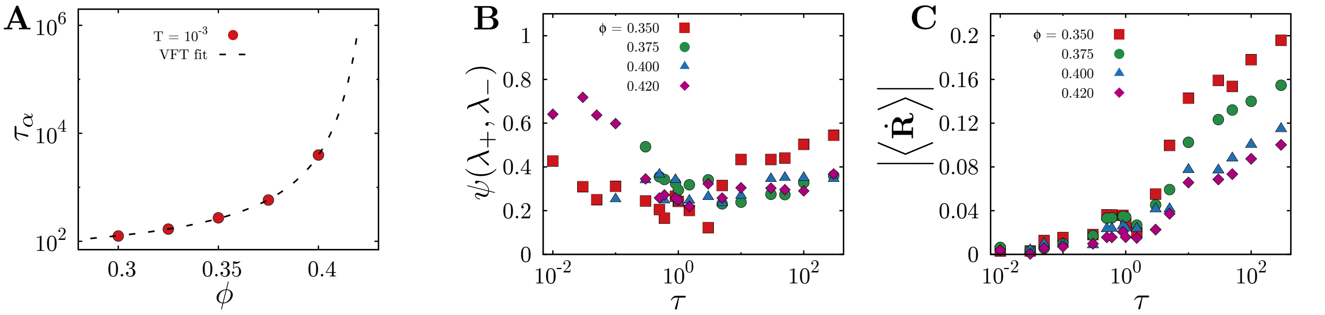

FIG. Supplementary Figure 2. **A** α-timescale of the passive medium as a function of the area fraction ϕ and the corresponding VFT fit. The value of τ_α for $\phi = 0.420$ in Fig. 3 is taken from the VFT fit. **B** Shape parameter (defined in Fig. 4**A** in main text) as a function of the persistence time τ of the active particle for small active forcing ($f = 0.5$). **C** Magnitude of the averaged velocity of the active particle with $f = 0.5$ in a passive medium of varying area fraction, as a function of τ.

Supplementary Note 4. LINEARISED HYDRODYNAMIC THEORY

We first write the full dynamics of the medium and the active particles, and their interplay. The medium is described by a local density field $\rho(\mathbf{r}, t)$ and a velocity field $\mathrm{v}(\mathbf{r}, t)$, and obeys, the continuity equation for ρ

$$\partial_t \rho + \nabla \cdot (\rho \mathrm{v}) = 0 \tag{S17}$$

and force-balance equation for v,

$$\Gamma \mathrm{v} = -B\rho\nabla\rho + f\sum_i \mathbf{n}_i(t)\,\delta^{(2)}(\mathbf{r} - \mathbf{R}_i(t)) \tag{S18}$$

In (S18) above, $B > 0$, since the pressure due to the medium opposes the local forcing from the active particles. The dynamics of the active particles in the dilute limit is given by

$$\gamma \dot{\mathbf{R}}_i = f\mathbf{n}_i - C\rho\nabla\rho\Big|_{at\ \mathbf{R}_i} \tag{S19}$$

$$\dot{\theta}_i = \xi_i(t) \tag{S20}$$

where γ is the drag on the active particle and C is the extent of back reaction of medium on the translational dynamics of the active particle. $\mathbf{n}_i \equiv (\cos\theta_i, \sin\theta_i)$, and the athermal orientational noise ξ_i has zero mean and is delta-correlated, $\langle \xi_i(t)\xi_j(t')\rangle = 2\tau^{-1}\delta_{ij}\delta(t - t')$.

In general, the frictions Γ, γ, the active force magnitude f and coefficients B and C could depend on ρ.

A. Single active particle moving through the compressible medium in $d = 2$

We will now restrict ourselves to a single active particle moving through the compressible medium,

$$\Gamma \mathrm{v} = -B\rho\nabla\rho + f\,\mathbf{n}(t)\,\delta^{(2)}(\mathbf{r} - \mathbf{R}(t)) \tag{S21}$$

where $\mathbf{R}(t)$ is the coordinate of the active particle. As the active particle moves through the medium, it creates density inhomogeneities, which relax over time; we will be interested in a linear analysis about the uniform density of the ploughed medium. In this limit, we take Γ and B to be independent of ρ, and ignore the back-reaction term C.

Plugging (S21) into the continuity equation (S17), we get

$$\partial_t \rho - B' \nabla \cdot \left(\rho^2 \nabla \rho\right) + f' \,\nabla \cdot \left(\rho \mathbf{n}\, \delta^{(2)}(\mathbf{r} - \mathbf{R}(t))\right) = 0 \tag{S22}$$

where we have absorbed Γ and redefined B', f' as B/Γ and f/γ respectively. To simplify analysis, we will assume that the persistence time of the active particle is large compared to the timescale of observation, i.e., $\mathbf{n}(t)$ is independent of time. After initial transients, the density of the medium can then be written in terms of the collective coordinate,

$$\rho(\mathbf{r}, t) = \rho(\mathbf{r} - \mathbf{R}(t))$$

Transforming coordinates to the co-moving frame of the active particle (without loss of generality, we take $\mathbf{n}$ to point along the $\hat{\mathrm{x}}$-direction),

$$\begin{aligned} u &= x - X(t) \\ w &= y \end{aligned} \tag{S23}$$

and so,

$$\begin{aligned} \partial_t &\longrightarrow \frac{\partial u}{\partial t}\partial_u = -\dot{X}(t)\partial_u = -v_0 \partial_u \\ \partial_x &\longrightarrow \frac{\partial u}{\partial x}\partial_u = \partial_u \\ \partial_y &\longrightarrow \frac{\partial w}{\partial y}\partial_w = \partial_w \end{aligned} \tag{S24}$$

where $v_0 \equiv f/\gamma$. The equation for the transformed density $\rho(u, w)$ in the co-moving frame now reads,

$$-\, v_0\, \partial_u \rho - B' \,\nabla \cdot \left(\rho^2 \nabla \rho\right) + f' \,\partial_u \left(\rho \delta^{(2)}(u, w)\right) = 0 \tag{S25}$$

where $\nabla \equiv (\partial_u, \partial_w)$.

We now express the local density as a small deviation (positive or negative) from the uniform ρ_0, and rewrite the above equation to linear order in this small deviation, i.e., we set $\rho \to \rho_0 + \rho$ in (S25), and linearise to get,

$$-\, v_0\, \partial_u \rho - B' \,\rho_0^2 \,\nabla^2 \rho + f' \,\partial_u \left((\rho_0 + \rho)\, \delta^{(2)}(u, w)\right) = 0 \tag{S26}$$

This linear equation can be solved by fourier transforming $(u, w) \to (q_u, q_w)$, and then evaluating the inverse fourier transform using contour integration. To proceed, we first note that,

$$\int_{-\infty}^{\infty} \int_{-\infty}^{\infty} (\rho_0 + \rho(u, w))\, \delta^{(2)}(u, w)\, \mathrm{e}^{-i q_u u} \mathrm{e}^{-i q_w w}\, du\, dw = \rho_0 + \rho(0, 0) \tag{S27}$$

which leads to,

$$-\, i v_0 q_u \widetilde{\rho} + B' \rho_0^2 q^2 \widetilde{\rho} + i f' q_u (\rho_0 + \rho(0, 0)) = 0 \tag{S28}$$

where $q^2 = q_u^2 + q_w^2$, from which we get,

$$\widetilde{\rho}(q_u, q_w) = \frac{i f' q_u (\rho_0 + \rho(0, 0))}{i v_0 q_u - B' \rho_0^2 q^2}\,. \tag{S29}$$

We now obtain $\rho(u,w)$, by inverse fourier transforming $\widetilde{\rho}(q_u,q_w)$,

$$\rho(u,w) = \frac{1}{4\pi^2}\int_{-\infty}^{\infty}\int_{-\infty}^{\infty}\widetilde{\rho}(q_u,q_w)\,\mathrm{e}^{iq_u u}\,\mathrm{e}^{iq_w w}\,dq_u\,dq_w$$

$$= -\frac{if(\rho_0+\rho(0,0))}{4\pi^2 B\rho_0^2}\int_{-\infty}^{\infty}\int_{-\infty}^{\infty}\frac{q_u}{q_u^2-\frac{iv_0}{B'\rho_0^2}q_u+q_w^2}\mathrm{e}^{iq_u u}\mathrm{e}^{iq_w w}\,dq_u\,dq_w \tag{S30}$$

$$= \int_{-\infty}^{\infty} I(u,q_w)\mathrm{e}^{iq_w w}\,dq_w \tag{S31}$$

where I is an integral over q_u, keeping q_w fixed,

$$I(u,q_w) = -\frac{if(\rho_0+\rho(0,0))}{4\pi^2 B\rho_0^2}\int_{-\infty}^{\infty}\frac{q_u}{q_u^2-\frac{iv_0}{B'\rho_0^2}q_u+q_w^2}\mathrm{e}^{iq_u u}\,dq_u \tag{S32}$$

We evaluate I in (S32) using contour integration. For this, we determine the simple poles $q_\pm$ of the integrand by factorization,

$$q_\pm = i\left(\xi^{-1}\pm\sqrt{\xi^{-2}+q_w^2}\right) \tag{S33}$$

where the length-scale ξ is given by,

$$\xi = \frac{2B'\rho_0^2}{v_0} \equiv \frac{2B\gamma\rho_0^2}{f\Gamma} \tag{S34}$$

when we restore the original parameter definitions.

Note that the $\operatorname{sgn}\operatorname{Im}(q_+) = +1$ and $\operatorname{sgn}\operatorname{Im}(q_-) = -1$. Thus we close the contour of integration in the upper half plane around q_+ when $u>0$, and in the lower half plane around q_- when $u<0$.

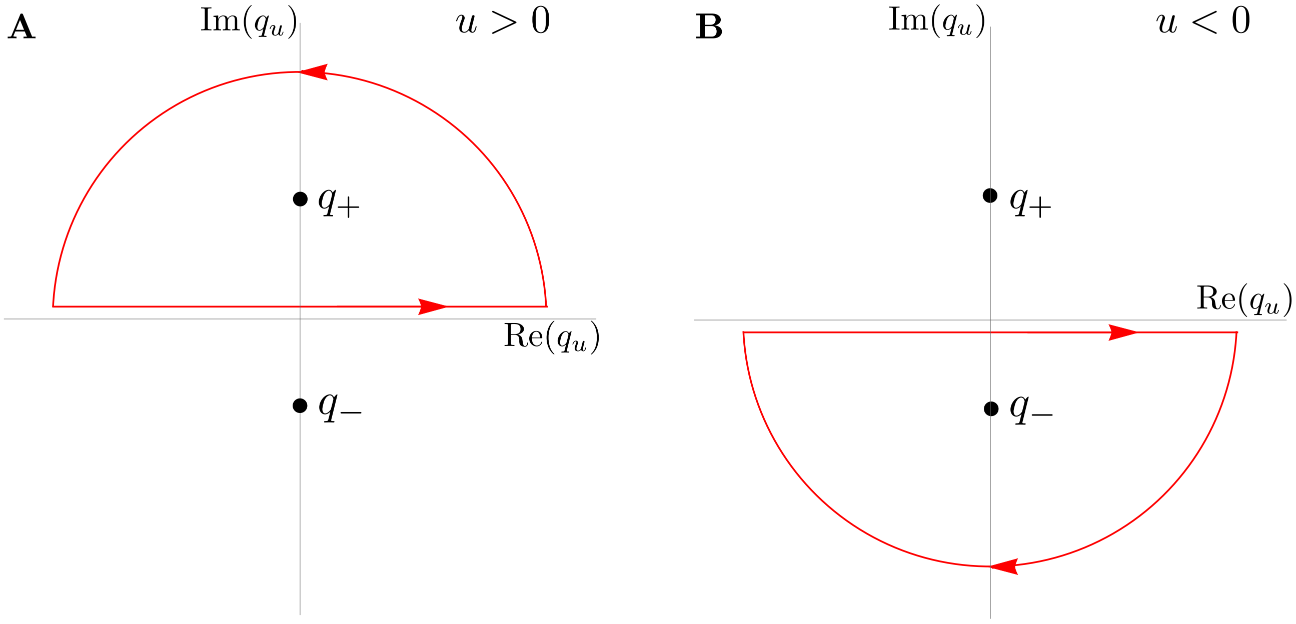

FIG. Supplementary Figure 3. Choice of contours for **A** $u>0$ and **B** $u<0$.

Using, $q_+ - q_- = 2i\sqrt{\xi^{-2}+q_w^2}$, we can evaluate the residues,

$$Res(q_+) = \frac{q_+}{q_+-q_-}\mathrm{e}^{iq_+u}$$

$$= \frac{1}{2}\left[1+\frac{\xi^{-1}}{\sqrt{\xi^{-2}+q_w^2}}\right]\mathrm{e}^{-\left(\xi^{-1}+\sqrt{\xi^{-2}+q_w^2}\right)u} \tag{S35}$$

and

$$Res(q_-) = \frac{q_-}{q_- - q_+} \mathrm{e}^{iq_- u}$$
$$= \frac{1}{2}\left[1 - \frac{\xi^{-1}}{\sqrt{\xi^{-2}+q_w^2}}\right] \mathrm{e}^{-\left(\xi^{-1}-\sqrt{\xi^{-2}+q_w^2}\right)u} \tag{S36}$$

The integral I (S32) can now be read out. For $u > 0$,

$$I(u, q_w) = -\frac{if(\rho_0 + \rho(0,0))}{4\pi^2 B\rho_0^2} 2\pi i\, Res(q_+)$$
$$= \frac{f(\rho_0 + \rho(0,0))}{4\pi B\rho_0^2}\left[1 + \frac{\xi^{-1}}{\sqrt{\xi^{-2}+q_w^2}}\right] \mathrm{e}^{-\frac{u}{\xi}} \mathrm{e}^{-u\sqrt{\xi^{-2}+q_w^2}} \tag{S37}$$
$$\tag{S38}$$

For $u < 0$,

$$I(u, q_w) = -\frac{if(\rho_0 + \rho(0,0))}{4\pi^2 B\rho_0^2} (-2\pi i)\, Res(q_-)$$
$$= -\frac{f(\rho_0 + \rho(0,0))}{4\pi B\rho_0^2}\left[1 - \frac{\xi^{-1}}{\sqrt{\xi^{-2}+q_w^2}}\right] \mathrm{e}^{-\frac{u}{\xi}}\ \mathrm{e}^{u\sqrt{\xi^{-2}+q_w^2}} \tag{S39}$$

Defining

$$\mathcal{D}(u) = \frac{f(\rho_0 + \rho(0,0))}{4\pi B\rho_0^2} \mathrm{e}^{-u/\xi} \tag{S40}$$

we can write the above expressions in compact notation

$$I(u^\pm, q_w) = \pm\mathcal{D}(u^\pm)\left[1 \pm \frac{\xi^{-1}}{\sqrt{\xi^{-2}+q_w^2}}\right] \mathrm{e}^{\mp u^\pm\sqrt{\xi^{-2}+q_w^2}} \tag{S41}$$

where $u^\pm$ denotes positive and negative u, respectively. Note that the function in (S41) is even in q_w. We now evaluate the integral in (S31),

$$\rho(u^\pm, w) = \pm\mathcal{D}(u^\pm)\int_{-\infty}^{\infty}\left[1 \pm \frac{\xi^{-1}}{\sqrt{\xi^{-2}+q_w^2}}\right] \mathrm{e}^{\mp u^\pm\sqrt{\xi^{-2}+q_w^2}}\ \mathrm{e}^{iq_w w}\, dq_w$$
$$= \pm 2\mathcal{D}(u^\pm)\int_0^{\infty}\left[1 \pm \frac{\xi^{-1}}{\sqrt{\xi^{-2}+q_w^2}}\right] \mathrm{e}^{\mp u^\pm\sqrt{\xi^{-2}+q_w^2}}\ \cos(wq_w)\, dq_w$$
$$= \pm 2\mathcal{D}(u^\pm)\left[\int_0^{\infty} \mathrm{e}^{\mp u^\pm\sqrt{\xi^{-2}+q_w^2}}\ \cos(wq_w)\, dq_w \pm \xi^{-1}\int_0^{\infty}\frac{\mathrm{e}^{\mp u^\pm\sqrt{\xi^{-2}+q_w^2}}}{\sqrt{\xi^{-2}+q_w^2}} \cos(wq_w)\, dq_w\right] \tag{S42}$$

From Erdelyi [12] – Integral Transform Table (1.4.26),

$$\int_0^{\infty} \mathrm{e}^{-u\sqrt{\xi^{-2}+q_w^2}}\ \cos(wq_w)\, dq_w \tag{S43}$$
$$= \mathcal{I}_1(u, w) \quad \text{for } u, w > 0$$
$$= \frac{u}{\xi}\left(u^2 + w^2\right)^{-\frac{1}{2}} K_1\left[\xi^{-1}\left(u^2 + w^2\right)^{\frac{1}{2}}\right] \tag{S44}$$

From Erdelyi [12] – Integral Transform Table (1.4.27),

$$\int_0^{\infty}\frac{\mathrm{e}^{-u\sqrt{\xi^{-2}+q_w^2}}}{\sqrt{\xi^{-2}+q_w^2}} \cos(wq_w)\, dq_w = \mathcal{I}_2(u, w) \quad \text{for } u, w > 0$$
$$= K_0\left[\xi^{-1}\left(u^2 + w^2\right)^{\frac{1}{2}}\right] \tag{S45}$$

where K_0 and K_1 are the modified Bessel functions of the second kind.

Therefore, for the whole range of u,

$$\rho(u,w) = 2\mathcal{D}(u)\left[\mathcal{I}_1(u,w) + \frac{\mathcal{I}_2(u,w)}{\xi}\right] \tag{S46}$$

where,

$$\begin{aligned}
\mathcal{I}_1(u,w) &= \frac{u/\xi}{\sqrt{u^2+w^2}} K_1\left[\frac{(u^2+w^2)^{\frac{1}{2}}}{\xi}\right] \\
\mathcal{I}_2(u,w) &= K_0\left[\frac{(u^2+w^2)^{\frac{1}{2}}}{\xi}\right] \\
\mathcal{D}(u) &= \frac{(\rho_0+\rho(0,0))f}{2\pi v_0 \xi \Gamma} \mathrm{e}^{-\frac{u}{\xi}} .
\end{aligned} \tag{S47}$$

The fore-aft asymmetry of the density profile is most apparent when we set $w = 0^+$. In this case, for $u > 0$,

$$\rho(u,0^+) = \frac{(\rho_0+\rho(0,0))f}{\pi v_0 \xi^2 \Gamma} \mathrm{e}^{-\frac{u}{\xi}} \left[K_1\left(\frac{u}{\xi}\right) + K_0\left(\frac{u}{\xi}\right)\right] \quad \text{and} \tag{S48}$$

for $u < 0$, noting that $\mathcal{I}_1(u,0^+) = -K_1\left(\frac{|u|}{\xi}\right)$,

$$\rho(u,0^+) = -\frac{(\rho_0+\rho(0,0))f}{\pi v_0 \xi^2 \Gamma} \mathrm{e}^{-\frac{u}{\xi}} \left[K_1\left(\frac{|u|}{\xi}\right) - K_0\left(\frac{|u|}{\xi}\right)\right] . \tag{S49}$$

Now using the asymptotic expansions [13]

$$K_0(z) \sim \sqrt{\frac{\pi}{2|z|}} e^{-z}\left(1 - \frac{1}{8z} + \dots\right) \tag{S50}$$

and

$$K_1(z) \sim \sqrt{\frac{\pi}{2|z|}} e^{-z}\left(1 + \frac{3}{8z} + \dots\right) \tag{S51}$$

for large z, we see immediately that the excess density profile in the moving frame is fore-aft asymmetric. While the profile decays exponentially following a pile up in the region $u > 0$, there is a slower power-law decay in the region $u < 0$, going as $|u|^{-3/2}$.

In a similar way, we obtain the density profile in the direction transverse to the direction of motion,

$$\rho(0^+,w) = \frac{(\rho_0+\rho(0,0))f}{\pi v_0 \xi^2 \Gamma} K_0\left(\frac{|w|}{\xi}\right) \tag{S52}$$

B. Two active particles moving through the compressible medium in $d = 2$

We will now be interested in how two active particles at $\mathbf{R}_1, \mathbf{R}_2$, moving through the compressible medium, affect each others dynamics. Let us again, take the limit of large persistence time - in this limit the orientations $\mathbf{n}_1$ and $\mathbf{n}_2$ are time independent. To $\mathcal{O}(\rho)$, these particles, individually, leave a time dependent asymmetric wake described by $\rho(u,w,t)$ in (S46)

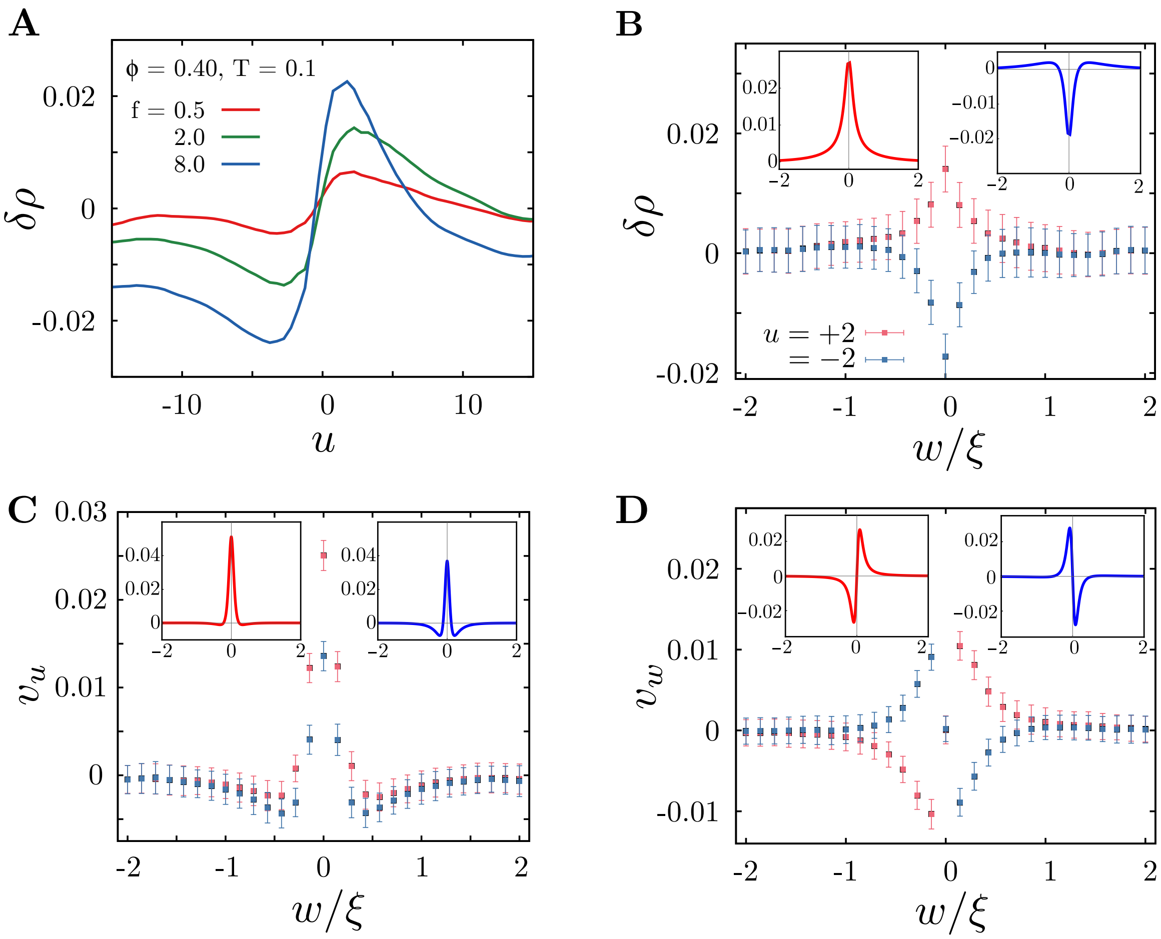

FIG. Supplementary Figure 4. **A** Excess density profile $\delta\rho$ in the frame of the moving active particle obtained from the simulations at $w = 0$, for different active forces, f, and $\phi = 0.40$. The decay length scale of the density wake (ξ) decreases as f increases. **B-D** The excess density, u-velocity and w-velocity profiles (for $\phi = 0.45$, $T = 0.1$) for $\frac{u}{\xi} \to 0^+$ (red), and $\frac{u}{\xi} \to 0^-$ (blue), as a function of $\frac{w}{\xi}$, with the predictions of the linear hydrodynamic theory in the inset.

To compute the back reaction of this wake on the movement of the active particles, we need to include terms to $\mathcal{O}(\rho)$ in (S20). Thus,

$$\gamma\dot{\mathbf{R}}_1 = f\mathbf{n}_1 - C\rho_0\nabla\rho\Big|_{\text{self}_1} - C\rho_0\nabla\rho\Big|_{1\leftarrow 2} \tag{S53}$$

$$\gamma\dot{\mathbf{R}}_2 = f\mathbf{n}_2 - C\rho_0\nabla\rho\Big|_{\text{self}_2} - C\rho_0\nabla\rho\Big|_{2\leftarrow 1} \tag{S54}$$

These equations may be cast in terms of the relative coordinate $\mathbf{R}_{rel} = \mathbf{R}_1 - \mathbf{R}_2$ and the centre of mass $\mathbf{R}_{cm} = (\mathbf{R}_1 + \mathbf{R}_2)/2$. We look at the following scattering geometries and initial conditions -

1. **Active particles 1 and 2 oriented along the fixed $\hat{\mathbf{x}}$ direction ($\mathbf{n}_1 = \mathbf{n}_2 = \hat{\mathbf{x}}$), with initial positions along the x-axis (particle 1 ahead of particle 2)**

   Note that the two active particles are identical, and so their self contributions *to this order* are the same. Therefore, in terms of the relative coordinate $X_{rel} = X_1 - X_2 > 0$ and the centre of mass $X_{cm} = (X_1 + X_2)/2$, (S53), (S54) can be recast as,

$$\gamma\dot{X}_{rel} = -C\rho_0\left(\partial_x\rho\Big|_{1\leftarrow 2} - \partial_x\rho\Big|_{2\leftarrow 1}\right) \tag{S55}$$

   and

$$\gamma\dot{X}_{cm} = f - C\rho_0\partial_x\rho\Big|_{\text{self}} - \frac{C\rho_0}{2}\left(\partial_x\rho\Big|_{1\leftarrow 2} + \partial_x\rho\Big|_{2\leftarrow 1}\right) \tag{S56}$$

We focus on the relative coordinate X_{rel}. The effect of particle 2 on 1 can be obtained from (S48)

$$\partial_u \rho\Big|_{1\leftarrow 2} = \frac{A}{\xi}\partial_z\left[e^{-z}[K_1(z)+K_0(z)]\right] \tag{S57}$$

The effect of particle 1 on 2 is obtained from (S49),

$$\partial_u \rho\Big|_{2\leftarrow 1} = \frac{A}{\xi}\partial_z\left[e^{z}[K_1(z)-K_0(z)]\right] \tag{S58}$$

where $A = \frac{f(\rho_0+\rho(0,0))}{\pi v_0 \xi^2 \Gamma}$ and $z = \frac{|u|}{\xi}$
Since the two active particles are identical, $\rho_1 = \rho_2 = \rho$, and so

$$\begin{aligned}\dot{X}_{rel} &= -\frac{C\rho_0 A}{\gamma\xi}\partial_z\left[e^{-z}[K_1(z)+K_0(z)] - e^{z}[K_1(z)-K_0(z)]\right]\Bigg|_{z=X_{rel}/\xi} \\ &= -\frac{C\rho_0 A}{2\gamma\xi}\left[e^{-z}\left\{3(e^{2z}-1)K_0(z) - 4(e^{2z}+1)K_1(z) + (e^{2z}-1)K_2(z)\right\}\right]\Bigg|_{z=X_{rel}/\xi}\end{aligned} \tag{S59}$$

This dynamical equation has a stable fixed point at $X^*_{\rm rel} \approx 1.5064\xi$ (indicated in the inset of Fig. 7C). This means that, according to this linearised 2-particle theory, there will be a bound 2-particle state, *i.e.*, eventually both particles will move forward keeping the same relative distance.

In the limit $z \to \infty$, the equation for the dynamics of X_{rel} becomes

$$\dot{X}_{rel} \approx -\frac{3}{4}\sqrt{\frac{\pi}{2}}\frac{C\rho_0 A}{\gamma\xi}\left(\frac{X_{rel}}{\xi}\right)^{-5/2} \tag{S60}$$

Similarly, for the motion of the centre of mass, we have,

$$\dot{X}_{cm} \approx \frac{f}{\gamma}\hat{\rm x} + \frac{3}{8}\sqrt{\frac{\pi}{2}}\frac{C\rho_0 A}{\gamma\xi}\left(\frac{X_{rel}}{\xi}\right)^{-5/2}\,. \tag{S61}$$

(S60) together with (S59) implies that if the two particles start from an initial separation $X_{\rm rel} \gg \xi$, the separation decreases with time with an initially increasing speed of approach, which, over time, decreases and the particles, eventually, move with the same speed, being bound to each other at a finite separation, $X^*_{\rm rel}$. This is a signature of *nonreciprocal sensing*.

We may extract the scaling behaviour from the dominant term in (S60). When $X_{\rm rel} \gg \xi$, one may obtain,

$$X_{rel} \propto |t - t_*|^{2/7}\,, \tag{S62}$$

as stated in the main text.

2. **Active particles 1 and 2 oriented along the fixed $\hat{\rm x}$ direction, with initial positions along the y-axis (particle 1 to the left of particle 2)**

$$\begin{aligned}\gamma\dot{Y}_{rel} &= -C\rho_0\left(\partial_y\rho\Big|_{1\leftarrow 2} - \partial_y\rho\Big|_{2\leftarrow 1}\right) \\ &= -2C\rho_0\partial_y\rho\Big|_{1\leftarrow 2}\end{aligned} \tag{S63}$$

and

$$\gamma \dot{Y}_{cm} = f - C\rho_0 \partial_y \rho\Big|_{\text{self}} - \frac{C\rho_0}{2}\left(\partial_y \rho\Big|_{1\leftarrow 2} + \partial_y \rho\Big|_{2\leftarrow 1}\right) \tag{S64}$$

The symmetry of the problem ensures that the last term in (S64) will be zero. To evaluate Y_{rel}, we compute

$$\begin{aligned}\partial_y \rho\Big|_{1\leftarrow 2} &= \partial_w \Big[- AK_0(\frac{w}{\xi})\Big] \\ &= \frac{A}{\xi}\partial_z \Big[- K_0(z)\Big]\Big|_{z=Y_{rel}/\xi} \\ &= \frac{A}{\xi}K_1(\frac{Y_{rel}}{\xi})\end{aligned} \tag{S65}$$

For $\frac{Y_{\text{rel}}}{\xi} \to \infty$

$$\partial_y \rho\Big|_{1\leftarrow 2} = \frac{A}{\xi}\sqrt{\frac{\pi}{2}} e^{-\frac{Y_{rel}}{\xi}} \left(\frac{Y_{rel}}{\xi}\right)^{-1/2} \tag{S66}$$

and for $\frac{Y_{rel}}{\xi} \to 0$,

$$\partial_y \rho\Big|_{1\leftarrow 2} = \frac{A}{\xi}\left(\frac{Y_{rel}}{\xi}\right)^{-1} \tag{S67}$$

(S65), when substituted in (S63), gives

$$\dot{Y}_{rel} = -\frac{2C\rho_0 A}{\gamma\xi}K_1(\frac{Y_{rel}}{\xi}) \tag{S68}$$

Unlike in Case (1), particles far separated along y do not feel a dynamical attractive force. However, if the y-distance between particles is of order ξ, then they will feel an effective attraction, which will make their trajectories *converge* towards each other. At late times, when $Y_{rel} \ll \xi$, one may obtain the scaling behaviour

$$Y_{rel} \propto |t - t_*|^{1/2} \tag{S69}$$

Supplementary Note 5. LINEARISED HYDRODYNAMICS THEORY IN $d = 1$: DENSITY PROFILES

The linearised theory shows that the excess density in $d = 1$ is very different from higher dimensions, because of the absence of a back flow in the medium. We will verify this against exact numerical solutions of the nonlinear equations in $d = 1$.

We first recall the linearised equations written in the comoving coordinate u of the motile particle,

$$-\, v_0\, \partial_u \rho - B'\, \rho_0^2\, \partial_u^2 \rho + f'\, \partial_u \left((\rho_0 + \rho)\, \delta(u) \right) = 0 \tag{S70}$$

As before, the solution is obtained by Fourier transformation,

$$\widetilde{\rho}(q_u) = \frac{if' q_u(\rho_0 + \rho(0,0))}{iv_0 q_u - B'\rho_0^2 q_u^2}\,. \tag{S71}$$

which upon inverse fourier transforming gives,

$$\rho(u) = \frac{1}{2\pi}\int_{-\infty}^{\infty} \widetilde{\rho}(q_u)\,\mathrm{e}^{iq_u u}\,dq_u$$
$$= -\frac{if(\rho_0+\rho(0,0))}{2\pi B\rho_0^2}\int_{-\infty}^{\infty}\frac{\mathrm{e}^{iq_u u}}{q_u - \frac{iv_0}{B'\rho_0^2}}\,dq_u \tag{S72}$$

The integrand has a simple pole at

$$q_+ = \frac{iv_0}{B'\rho_0^2} = i\xi^{-1} \tag{S73}$$

where the length scale $\xi = B\rho_0^2/(v_0\Gamma)$. Note that the $\operatorname{sgn}\operatorname{Im}(q_+) = +1$.

For u > 0, the integrand will tend to zero only when $\operatorname{Im}(q_u) > 0$. For u < 0, the integrand will tend to zero only when $\operatorname{Im}(q_u) < 0$. Thus, the contour of the integral should traverse the upper half plane around q_+ when u > 0, and in the lower half plane when u < 0.

This immediately shows that the excess density

$$\rho(u) = \frac{f(\rho_0+\rho(0,0))}{B\rho_0^2}\mathrm{e}^{-\frac{u}{\xi}} \quad \text{for } u > 0$$
$$= 0 \quad \text{for } u < 0 \tag{S74}$$

Unlike the 2d density profile, there is no trailing wake in $d = 1$.

Supplementary Note 6. NUMERICAL SOLUTION OF NONLINEAR HYDRODYNAMIC EQUATIONS IN $d = 1$

We numerically solve the 1-dimensional non-linear equations corresponding to (S17)- (S20), with no noise. We have used finite volume discretization with an exponential scheme [14] for the convective flux as implemented in NIST-FiPy [15]. For the stability of the numerical scheme, we consider a diffusion term in the density field evolution $D\partial_x^2\rho$ with a very small diffusion constant ($D \sim 10^{-3}$). According to the exponential scheme, the convective problem is discretized and solved in each grid using an approximate form of the convective transport with constant coefficients $\partial_x(\mathrm{v}\rho - D\partial_x\rho) = \partial_x J_x = 0$. Thus, the local (over a grid) analytical solution for the density becomes exponential. This analytical expression is then used to estimate the flux. The flux at the interface of the ith and $(i-1)^{th}$ grid in this scheme is given by

$$J_x = \frac{D}{\Delta x}\left(B\rho^{i-1} - A\rho^i\right)$$
$$A = \frac{Pe}{e^{Pe}-1}$$
$$B = A + Pe\,, \tag{S75}$$

where $Pe = \frac{\mathrm{v}\Delta x}{D}$ is the Péclet number and Δx is the grid spacing. This scheme guarantees positive solutions and has low diffusive error as the flux is formulated using the exact solution. A first order temporal discretization was used in combination with a *sweep* between each iteration using Newton's method. Numerical solution of the nonlinear equation shows perfect agreement with the linear theory for $d = 1$ and that the density profile has the exponential decay in the front without any trailing wake, consistent with the linear theory (Fig. Supplementary Figure 5A). We also capture the dynamical transition (Fig. Supplementary Figure 5B) of the active particle with increasing propulsion force f.

Supplementary Note 7. FINITE-SIZE EFFECTS

We observed a strong finite size effect while analyzing the simulation data. This is because there is an intrinsic length scale set by ξ whose effect on the asymptotic decay can be corrupted due to the effects of periodic boundaries if the system size is not sufficiently large. This implies that one should be careful while comparing the theoretical

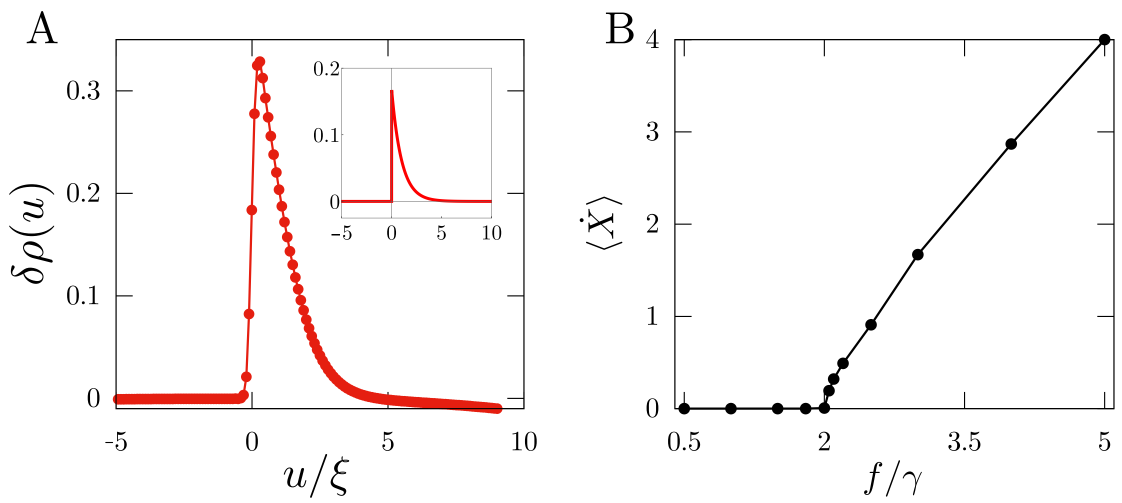

FIG. Supplementary Figure 5. **(A)** Density profile in the co-moving frame obtained from an 'exact' numerical solution of the nonlinear equation in $d = 1$. Inset shows analytical result from the linear theory. Parameter values used are $B/\Gamma = 6$, $C/\gamma = 10$ and $f/\gamma = 2$. **(B)** Dynamical transition of the active particle as a function of propulsion force f. This behaviour is also observed in the simulations in 2-dimensions (Fig. 4C in main text). The parameter values used are $B/\Gamma = 8.6$ and $C/\gamma = 25.5$. The space and time discretisation used are $dx = 0.5$ and $dt = 0.05$, respectively.

predictions of the linearised hydrodynamic theory with simulations. For instance, at a system size of $L_0 = 45\sigma_{AA}$ with $N = 1260$ particles (shown in red in Fig. Supplementary Figure 6), the decay of the density wake shows a $(u/\xi)^{-1/2}$ scaling (Fig. Supplementary Figure 6**B**). It is only at a sufficiently large system size $L_0 = 90\sigma_{AA}$ with $N = 5040$ particles (shown in blue in Fig. Supplementary Figure 6), that we obtain a $(u/\xi)^{-3/2}$ scaling (Fig. Supplementary Figure 6**C**), that is consistent with the linearised hydrodynamic theory.

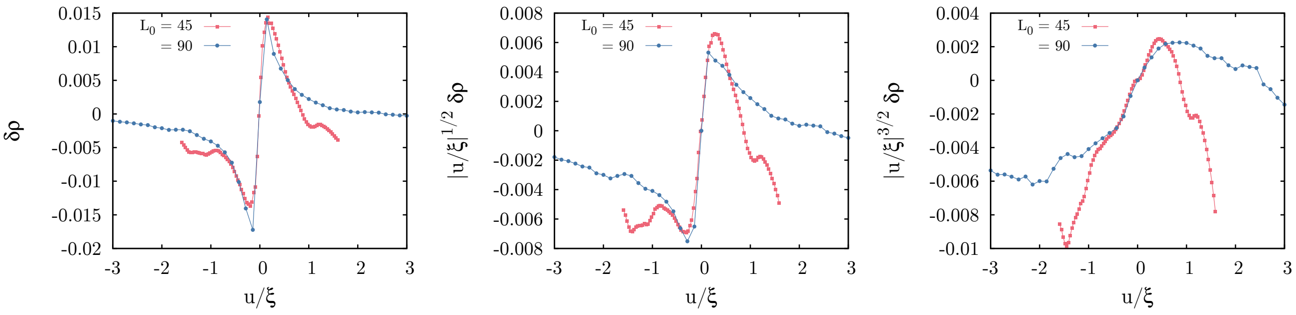

FIG. Supplementary Figure 6. Comparison of density profiles at $w = 0$ from simulations performed on two system sizes, $L_0 = 45$ (red) and $L_0 = 90$ (blue). **(A)** is the raw density profile as a function of u/ξ, while **(B)** and **(C)** are the same profiles scaled by $|u/\xi|^{-1/2}$ and $|u/\xi|^{-3/2}$, showing a flattening of profile for $u/\xi < 0$, only for the larger system where the decay is $|u/\xi|^{-3/2}$.

---

* Equal Contribution
† shashi@ncbs.res.in
‡ madan@ncbs.res.in

[1] W. Kob and H. C. Andersen, Phys. Rev. E **51** 4626-4641 (1995).
[2] R. Brüning, D. A. St-Onge, S. Patterson, and W. Kob, *Glass transitions in one-, two-, three-, and four-dimensional binary Lennard-Jones systems*, J. Phys. Condens. Matter , **21**, 035117 (2008).
[3] M. C. Marchetti, J.-F. Joanny, S. Ramaswamy, T. B. Liverpool, J. Prost, Madan Rao and R. Aditi Simha, *Hydrodynamics of Soft Active Matter*, Rev. Mod. Phys. **85**, 1143 (2013).
[4] Y. Fily and M. C. Marchetti, *Athermal phase separation of self-propelled particles with no alignment*, Phys. Rev. Lett., **108**, 235702 (2012).

[5] S. C. Takatori and J. F. Brady, *Towards a thermodynamics of active matter*, Phys. Rev. E, **91**, 032117 (2015).
[6] D. Levis, J. Codina and I. Pagonabarraga, *Active Brownian equation of state: metastability and phase coexistence*, Soft Matter, **13**, 8113 (2017).
[7] M. E. Cates and J. Tailleur, *Motility-induced phase separation*, Annu. Rev. Condens. Matter Phys., **6**, 219 (2015).
[8] R. Mandal, P. J. Bhuyan, M. Rao and C. Dasgupta, *Active fluidization in dense glassy systems*, Soft Matter **12**, 6268-6276 (2016).
[9] B. Smit and D. Frenkel, *Understanding Molecular Simulation: From Algorithms to Applications*, (Academic Press, 1996).
[10] J. C. Butcher, *Numerical Methods for Ordinary Differential Equations*, (New York: John Wiley & Sons, 2008)
[11] T. G. Mason, *Estimating the viscoelastic moduli of complex liquids using the generalized Stokes-Einstein equation*, Rheologica Acta **39**, 371–378 (2000).
[12] A. Erdelyi, (Ed). *Tables of Integral Transforms.* vol. **1**, (McGraw-Hill, 1954).
[13] M. Abramowitz, I. A. Stegun and R.H. Romer, *Handbook of mathematical functions with formulas, graphs, and mathematical tables*, American Association of Physics Teachers, 1988.
[14] D. N. de G. Allen, and R. V. Southwell, *Relaxation methods applied to determine the motion, in two dimensions, of a viscous fluid past a fixed cylinder*, The Quarterly Journal of Mechanics and Applied Mathematics **8** 129-145 (1955).
[15] J. E. Guyer, D. Wheeler and J. A. Warren, *FiPy: Partial differential equations with Python*, Computing in Science & Engineering **11**, 6-15 (2009).
